# IRP1/ARID3A complex promotes pancreatic cancer chemoresistance by suppressing CYGB-related ferroptosis

**DOI:** 10.1016/j.gendis.2025.101866

**Published:** 2025-09-24

**Authors:** Zeru Li, Cheng Qin, Bangbo Zhao, Tianyu Li, Yutong Zhao, Lirui Huang, Haoyu Shi, Yiping Xie, Yutong Yan, Xiangyu Zhang, Weibin Wang

**Affiliations:** Department of General Surgery, State Key Laboratory of Complex Severe and Rare Diseases, Peking Union Medical College Hospital, Chinese Academy of Medical Sciences and Peking Union Medical College, Beijing 100730, China

**Keywords:** Chemosensitivity, Chromatin accessibility, Epigenetics, Ferroptosis, Pancreatic cancer

## Abstract

Ferroptosis, a unique modality of regulated cell death, has become an emerging strategy for tumor therapy. Multiple cellular pathways, including redox homeostasis, iron handling, epigenetic regulation, and metabolic changes, could mediate ferroptosis. Here, we demonstrate that high expression of iron-responsive element binding protein (IRP1)/A + T rich interaction domain protein 3a (ARID3A) inhibits ferroptosis and enhances chemoresistance of pancreatic cancer cells via handling the promoter region of a ferroptosis gene, cytoglobin (CYGB). Mechanistically, the high level of iron leads to nuclear translocation of IRP1 and ARID3A, thereby mediating ARID3A binding to the promoter region of CYGB and down-regulation of chromatin accessibility. The decrease of CYGB expression results in pancreatic cancer cell resistance to ferroptosis, which makes them more resistant to chemotherapy. Clinically, high expression of IRP1 and ARID3A associates with unsatisfactory chemotherapeutic response and poor survival of patients with pancreatic cancer. Our study highlights the role of IRP1/ARID3A complex as a chemotherapy target and its potential in the combined application of ferroptosis drugs.

## Introduction

Epigenomic alterations, including histone modification and chromatin remodeling, are present in human cancers and are now known to cooperate with genetic changes to drive the cancer phenotype. Chromatin is the carrier of genetic information and consists of DNA, histones, non-histone proteins, and a small amount of RNA.^1^ Chromatin and nucleosomes control the stability of the eukaryotic genome and regulate DNA processes such as transcription, replication, and repair. The dynamic structure of chromatin plays a key role in this regulatory function.^2^ Pancreatic cancer (PCa) is one of the most malignant cancers, with a 5-year survival rate of 12%.^3^ Although many studies have characterized the molecular subtypes of PCa, it is necessary to explore tumor-specific transcription factors and epigenetic abnormalities due to its high heterogeneity.

Ferroptosis is a form of regulated cell death driven by iron-dependent lipid peroxidation. This unique modality of cell death is regulated by multiple cellular pathways, including redox homeostasis, iron handling, mitochondrial activity, and amino acid metabolism, in addition to various signaling pathways relevant to disease.^4^ In recent years, the role of ferroptosis in PCa has been fully explained. For instance, the conditional depletion of glutathione peroxidase 4 (GPX4) in the pancreas or a high-iron diet accelerates the development of Kras G12D-driven pancreatic tumors in mice through the release of nuclear 8-hydroxydeoxyguanosine (8-OH-dG) by ferroptosis cells. 8-OH-dG activates the stimulator of interferon response CGAMP interactor 1 (STING1, also known as TMEM173) pathway in surrounding macrophages, thereby inducing the release of cytokines (*e.g.*, interleukin 6) to maintain the chronic inflammatory microenvironment of pancreatic tumorigenesis driven by Kras G12D.^5^ Basic helix-loop-helix family member e40 (BHLHE40)-sterol regulatory element binding transcription factor 1 (SREBF1)-stearoyl-CoA desaturase 1 (SCD1)-ferroptosis promotes PCa progression. Increasing chromatin accessibility in the related region leads to a significant up-regulation of BHLHE40 expression in PCa. BHLHE40 not only regulates the transcription of SREBF1, but also connects the enhancer and promoter regions of SREBF1 through liquid–liquid phase separation.^6^ At present, the commonly used chemotherapy schemes for PCa show different degrees of drug resistance. Exciting preclinical studies have shown that several drugs, such as artemisinin^7^ and zacetabine,^8^ can inhibit PCa by inducing ferroptosis, although they may have off-target effects. Several treatment options related to ferroptosis, such as gemcitabine (GEM) plus sulfasalazine and sorafenib plus sulfasalazine, have also been explored in PCa animal models. The goal of the study is to develop drugs that can be used clinically to regulate the ferroptosis pathway, which can kill PCa alone or in combination with other drugs.

Iron regulatory protein 1 (IRP1) is a bifunctional protein related to iron metabolism, and its activity depends on the presence of the 4Fe–4S cluster.^9^ IRP1 is a recognized cytoplasmic protein, but studies in drosophila models and mammals have shown that it can translocate to the nucleus under iron overload. With the fluctuation of intracellular iron concentration, IRP1 shifts between apo-IRP1 and holo-IRP1 forms. When iron levels are normal or overloaded, apo-IRP1 is repaired by 1,4-alpha-glucan branching enzyme (AGBE), CDGSH iron sulfur domain 2 (Cisd2), and ferritin, and harbors an iron-sulfur [4Fe–4S] cluster (holo-IRP1) and translocates to the nucleus.^10^^,^^11^

A + T rich interaction domain protein 3a (ARID3A) is a class of transcription factors rich in AT interaction domains. Its motif TTTAAT can bind to DNA and regulate gene transcription. It was first discovered in B cells and was initially named B cell IgH transcription regulatory factor. In B cells, ARID3A can alter IgH expression by regulating chromatin accessibility.^12^ In head and neck squamous cell carcinoma cells, ARID3A is translated and synthesized by let-7 post-transcriptional regulation in the cytoplasm, which can be introduced into the nucleus through importin 9 (IPO9), and form transcriptional complexes with ARID3B and lysine demethylase 4C (KDM4C). By down-regulating the level of histone 3 lysine 9 trimethylation (H3K9me3) modification in the promoter regions of high mobility group AT-hook 2 (HMGA2), c-Myc, H-RAS, and other genes, it promotes the expression of these let-7 downstream stem genes, thus inducing the phenotype of tumor stem cells.^13^

Cytoglobin (CYGB) is a member of the globulin family with high phylogenetic and structural similarity to myoglobin, hemoglobin, and neuroglobin. CYGB is widely present in cells and exerts a broader range of roles than myoglobin, hemoglobin, and neuroglobin. In normal cells, CYGB exhibits respiratory roles through oxygen storage, reactive oxygen species (ROS) regulation, and antifibrotic activity.^14^ In recent years, CYGB has been found to have an important role in tumor progression. In breast cancer, CYGB inhibits glucose transporter 1 (GLUT1) and hexokinase 2 (HXK2) in a p53-dependent or non-dependent manner, thereby inhibiting glycolysis and cell proliferation.^15^ In colon cancer, CYGB activates p53 and yes-associated protein 1 (YAP1) to promote lipid peroxidation and ferroptosis.^16^

Here, we firstly present that IRP1 shows higher expression in cell lines resistant to ferroptosis inducers (Hemin and Erastin used in our study) and leads to chemoresistance of PCa cells. Then we performed co-immunoprecipitation and mass spectrometry to explore the downstream transcription factor of IRP1. In the presence of iron overload, IRP1 exhibits as holo-IRP1 ([4Fe–4S] cluster-containing state), tightly binding to ARID3A and carrying ARID3A into the nucleus. As a transcription factor, ARID3A binds to the promoter region of CYGB, while holo-IRP1 reduces the chromatin accessibility of this region. Together, IRP1/ARID3A complex inhibits CYGB transcription and suppresses ferroptosis of PCa cells. In clinical application, targeting the IRP1/ARID3A complex may provide a new strategy for PCa chemotherapy plus ferroptosis drug therapy.

## Materials and methods

### PCa cell lines

Six PCa cell lines were used in this research. All the cell lines were purchased from American Type Culture Collection (Virginia, USA). SW1990 was cultured in RPMI-1640 medium (Hyclone, USA). MIA-Paca2, PANC-1, Patu-8988, and T3M4 were cultured in DMEM-high glucose medium (Hyclone, USA). CFPAC was cultured in IMDM medium (Hyclone, USA). The culture mediumwas added with 10% fetal bovine serum.

### siRNA, plasmid, and lentivirus

The CMV enhancer-MCS-3flag-polyA-EF1A-zsGreen-sv40-puromycin vector (GeneChem, China) was used to overexpress IRP1, ARID3A, and CYGB. Gene-specific siRNAs and nonsense control were provided by Tsingke Biotechnology Co., Ltd., Beijing, China. The target two sequences for IRP1 knockdown were CCAGGAAAGAAATTCTTCAAT and CCTGCTGATCTTGTAATAGAT. The two sequences for CYGB knockdown were CCTCGGCCAAGCAGTACTT and AGTACTTCAGCCAGTTCAA. Stable knockdown or overexpression lentiviruses were provided by Shanghai Genechem Co., Ltd., China. The sequences of shRNA lentivirus against ARID3A were ACATCTACCTCAAATAACT and CTTACGAGGAGCAGTTTAA. PCa cells were cultured in 6-well plates, and Lipofectamine 3000 (Invitrogen, USA) was used for transfecting siRNA. PCa cells were selected with puromycin after viral transduction.

### Quantitative reverse transcription PCR

Total RNA was extracted by TRIzol regent (Invitrogen, USA) and reverse-transcribed to cDNA using a One Step SYBR® PrimeScripttm RT-PCR Kit (Vazyme, China) according to the manufacturer's instructions. The 2^−ΔΔCt^ method was used to quantify fold changes with normalization to *ACTB.* Detailed information on the primer sequences is shown in Table S1.

### Western blotting

Western blotting was conducted according to our previous study.^17^ The antibodies used in this study are listed in Table S2. Super-sensitive enhanced chemiluminescence assay kit (Beyotime, China) was used to show the immune response.

### Immunohistochemistry and immunohistofluorescence staining

Immunohistochemistry staining was performed using standard protocols. The tissue microarray (5 μM thickness), which includes 66 PCa tissues. The enrolled patients signed the informed consent form, and this study was approved by the ethical committees of Peking Union Medical College Hospital (I-23PJ2081). The protein levels of IRP1, ARID3A, and CYGB were measured on the tissue microarray. The specimens were scored as follows: staining intensity was scored 0 (negative), 1 (low), 2 (medium), and 3 (high), and staining extent was scored 0 (0% stained), 1 (1%–25% stained), 2 (26%–50% stained), and 3 (51%–100% stained). The staining score was equal to the proportion of tumor cells multiplied by the staining intensity. A staining score from 0 to 4 was defined as low expression, and 6–12 was defined as high expression. The nuclear fluorescence intensity and number of positive ARID3A and IRP1 were quantitatively evaluated using ImageJ. All the immunohistochemistry results were independently evaluated by two pathologists.

### Immunofluorescence

After treatment, PCa cells were seeded in a chambered cover-glass (Thermo Scientific, USA). After 24-h culture at 37 °C, 4% paraformaldehyde was added to fix the cells for 15 min. Subsequently, the cells were permeabilized with 1% Triton X-100 for 10 min. 5% bovine serum albumin was used to block at 37 °C for 1 h, and the samples were then incubated with anti-ARID3A or anti-IRP1 at 4 °C overnight. The next day, fluorophore-conjugated secondary antibodies (Proteintech, China) were used and incubated at room temperature in the dark for 2 h, and then stained with DAPI (ZSGB-BIO, China) for 2 min. Images were visualized by confocal fluorescence microscopy (AXR, Nikon).

### Cell cytotoxicity

Cells were seeded into 96-well plates, and 8 h later, GEM, GEM plus hemin, or GEM plus erastin was added (GEM concentration gradient: 0, 1 nM, 10 nM, 100 nM, 1 μM, 10 μM, 100 μM, and 1 mM). After being treated for 48 h, the cells were fixed with 10% trichloroacetic acid (20 min) and stained with 0.4% sulforhodamine B (30 min). Then, the stained cells were washed with 1% acetic acid repeatedly, and the dye was dissolved in 10 mM Tris-base. The optical density value at 562 nm was measured.

### RNA sequencing

#### *Cell-based RNA sequencing*

TRIzol was utilized to extract total RNA of PCa cells transfected with scrambled RNAi or RNAi against ARID3A. Each group was assigned three replicates, and the quality and quantity of RNA were controlled. RNA integrity was assessed using the RNA Nano 6000 Assay Kit of the Bioanalyzer 2100 system (Agilent Technologies, California, USA). Total RNA was used as input material for the RNA sample preparations. Differential expression analysis of the two groups was performed using the DESeq2 R package (1.20.0).

#### *Tissue-based RNA sequencing*

Three micrograms of total RNA were used for stranded RNA sequencing library preparation using KCTM Stranded mRNA Library Prep Kit for Illumina® (Catalog No. DR08402, Wuhan Seqhealth Co., Ltd., China) following the manufacturer's instructions. PCR products corresponding to 200–500 bp were enriched, quantified, and finally sequenced on the DNBSEQ-T7 sequencer (MGI Tech Co., Ltd., China) with the PE150 model. Raw sequencing data were first filtered by Trimmomatic (version 0.36), low-quality reads were discarded, and the reads contaminated with adaptor sequences were trimmed. Clean data were mapped to the reference human genome. Genes differentially expressed between groups were identified using the edgeR package (version 3.12.1). A *P*-value cutoff of 0.05 and fold-change cutoff of 2 were used to judge the statistical significance of gene expression differences.

### CUT&Tag library and sequencing

Cleavage under targets and tagmentation (CUT&Tag) libraries were conducted using the NovoNGS® CUT&Tag 4.0 High-Sensitivity Kit (for Illumina®) (Novoprotein, China). Briefly, PCa cells were incubated with activated NovoNGS ConA beads. Subsequently, the bead-bound cells were permeabilized, firstly incubated with a primary antibody against ARID3A, and then incubated with anti-rabbit antibody (Abcam, USA). Then, the diluted pAG-Tn5 adapter complex was added and subjected to a labeling reaction. The extracted DNA fragments were used for library preparation. After amplification, the libraries were sequenced by the Illumina NovaSeq 6000 platform.

### ATAC-sequencing library and sequencing

ATAC-sequencing (short for assay for transposase-accessible chromatin using sequencing) libraries were conducted using the Hyperactive ATAC-Seq Library Prep Kit for Illumina (Vazyme Biotech, China). Briefly, 1 × 10^5^ cells were lysed to isolate the nuclei. The nuclei were extracted and resuspended in a 50 μL Tn5 transposase reaction mixture. The product was then purified by QIAquick PCR Purification Kit (QIAGEN, Germany). PCR was performed for library amplification, and DNA clean beads were used to purify the PCR product. The libraries were then sequenced on an Illumina HiSeq X Ten system.

### Animal experiments

All the animal experiments were approved by the Institutional Animal Care and Use Committee in Peking Union Medical College Hospital (XHDW-2023-081). PCa cells were transfected with the aforementioned lentiviruses and subcutaneously injected into the right armpit region of 6-week-old BALB/c nude mice (Chinese Academy of Sciences, China) (5 × 10^6^ cells per mouse). After the palpable tumor formation, we measured tumor size once a week and calculated tumor volume (mm^3^ = 1/2 × length × width^2^). Five weeks later, the mice were euthanized, and the tumors were dissected for subsequent immunohistochemistry staining and analysis.

### Total ROS level

Cells were seeded in chambered cover-glass (Thermo Scientific, USA) at 1 × 10^4^ cells per well overnight. To detect ROS, a 10 mM 2′,7′-dichlorofluorescin diacetate (DCFH-DA) probe dissolved in dimethyl sulfoxide was prepared according to the instructions of the ROS test kit (Solarbio, China). DCFH-DA was added to the medium without phenol red to a final concentration of 10 μM and cultured at 37 °C for 30 min. Images were visualized by confocal fluorescence microscopy (AXR, Nikon). The fluorescence intensity was quantified with ImageJ software.

### Promoter targeting CRISPR editing

CRISPOR (http://crisp) was used to design the promoter targeting CRISPR Epigenetic editing short guide RNAs (sgRNAs) used in CRISPR interference/activation (CRISPRi/a). The targeting sequences were sgPRO-1-TCAATAGGGCTAGAGCCCGGAGG and sgPRO-2-TCCGGGCTCTAGCCCTATTGAGG. The pX330a-dCas9-KRAB (MiaoLingbio, China) and pX330a-dCas9-VP64 (MiaoLingbio, China) were transfected into PCa cells. T7 transcription kit (Novoprotein, China) for *in vitro* transcription was used to generate CRISPRi/a system in PCa cells. PCa cells stably expressing the dCas9-KRAB or pX330a-dCas9-VP64 fusion protein were transfected with the sgRNAs using Lipofectamine™ 3000 (Invitrogen, L3000015). After 48 h post-transfection, CYGB expression was assessed using quantitative reverse transcription PCR.

### Chromatin immunoprecipitation

The Pierce Magnetic ChIP kit (Thermo Scientific, USA) was used to detect the interaction between ARID3A and the promoter region of CYGB according to the manufacturer's instructions. PCa cells were collected after fixation with 4% formaldehyde. Then, the cells were lysed and the nucleic acids were degraded using micrococcal nuclease (MNase). Ultrasonic treatment was performed at 4 °C to break the chromatin into fragments. The chromatin-protein complex was incubated with 5 μg anti-IgG or anti-ARID3A (Proteintech, China). Ultimately, the DNA was purified using protein A/G magnetic beads. The results were analyzed by quantitative reverse transcription PCR.

### Dual luciferase reporter assay

Cells were plated on 6-well plates at 3 × 10^5^ cells per well. After 24 h of culture, the cells were co-transfected with the designed firefly luciferase reporter plasmids, Renilla luciferase plasmids, and corresponding empty vectors (Genechem, China). Firefly and Renilla fluorescence signals were detected 48 h after transfection using a dual luciferase reporter assay kit (YEASEN, Shanghai, China) by a fluorescence microplate reader.

### Co-immunoprecipitation assay

The IRP1 and ARID3A antibodies were used in the co-immunoprecipitation assays according to the manufacturer's instructions (Thermo Fisher, USA). Cells were harvested and lysed with lysis buffer directly on plates for 5 min. Meanwhile, 25 μL Pierce protein A/G beads were incubated with 8 μg antibody at room temperature for 15 min. Then, the protein lysate and the beads–antibody complex were mixed and incubated at room temperature for 1 h. Beads were washed two times with the wash buffer, and the products were then detected by Western blotting.

### Statistical analyses

GraphPad Prism 10 Software was used to generate graphs and statistical results. Unpaired Student's *t*-test, paired Student's *t*-test, or ANOVA was used to compare differences among groups. Overall survival curves were performed via the Kaplan–Meier method. All the experiments were repeated at least three times, and *P* values less than 0.05 were considered statistically significant.

## Results

### High levels of IRP1 correlate with iron tolerance and chemoresistance of PCa cells

**Figure 1:**
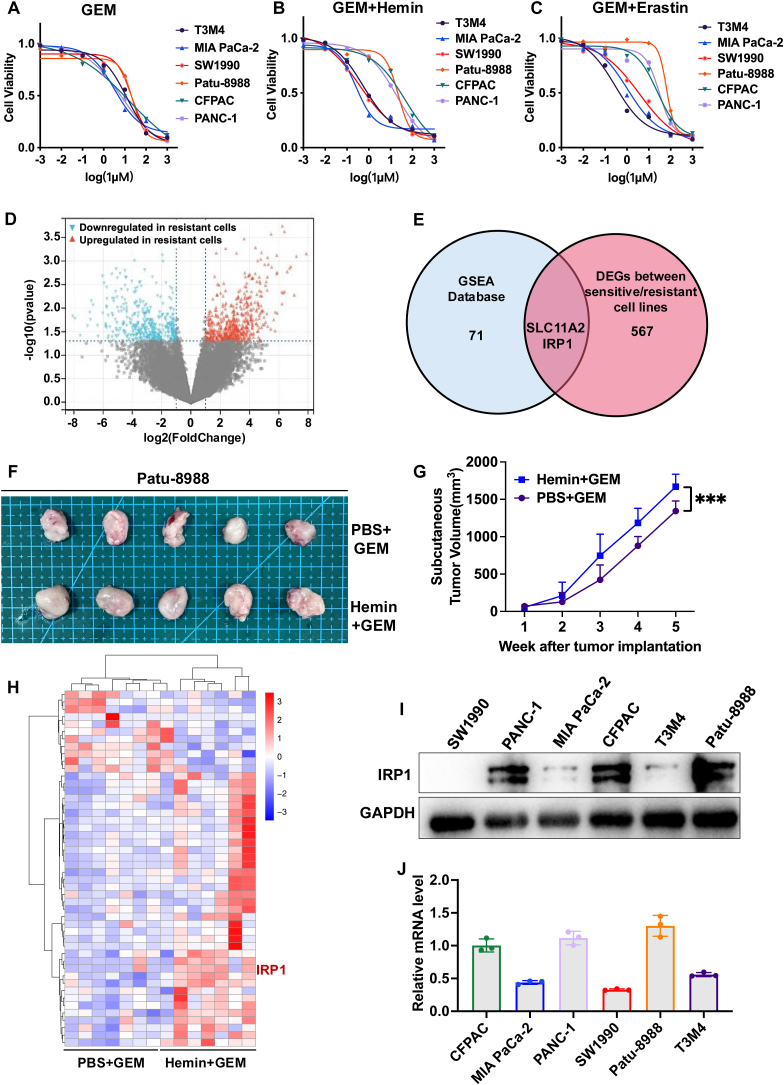
IRP1 is associated with ferroptosis and drug resistance in PCa cells. **(A)** IC50 curves of GEM in the six common PCa cell lines. **(B)** IC50 curves of GEM plus hemin in the six common PCa cell lines. **(C)** IC50 curves of GEM plus erastin in the six common PCa cell lines. **(D)** Volcano plot of the differentially expressed genes between ferroptosis inducer-resistant and ferroptosis inducer-sensitive cell lines. **(E)** The results of the intersection for differentially expressed genes and the gene set enrichment analysis (GSEA) database. **(F)** Patu-8988 cells were subcutaneously inoculated in nude mice and followed by injection with GEM only or GEM plus hemin. **(G)** Tumor growth curves of the two groups of nude mice (∗∗∗*P* < 0.001). **(H)** Heatmap of differentially expressed genes between GEM plus PBS and GEM plus hemin of xenograft tissue RNA sequencing. **(I)** IRP1 protein expression level of the six cell lines was measured by Western blotting. **(J)** IRP1 mRNA expression level of the six cell lines was measured by quantitative reverse transcription PCR. GEM, gemcitabine; PCa, pancreatic cancer; PBS, phosphate-buffered saline.

To explore the key genes of ferroptosis that lead to chemoresistance in PCa, we detected the drug resistance of six PCa cell lines to GEM, GEM plus hemin (20 μM), and GEM plus erastin (10 μM) and fitted the IC50 curves (Fig. 1A–C). The results showed that the addition of ferroptosis inducers significantly improved the chemoresistance of MIA PaCa-2, SW1990, and T3M4, while Patu-8988, PANC-1, and CFPAC1 were tolerant to the two ferroptosis inducers. Subsequently, we utilized the Cancer Cell Line Encyclopedia (CCLE) database to analyze the differentially expressed genes between cell lines tolerant and sensitive to ferroptosis inducers. The differentially expressed genes were intersected with the ferroptosis-related dataset from gene set enrichment analysis (GSEA) (M11074, GOBP_INTRACELLULAR_IRON_ION_HOMEOSTASIS, http://amigo.geneontology.org/amigo/term/GO:0006879). IRP1 and SLC11A2 were two key genes that caused PCa cell lines to tolerate ferroptosis inducers (Fig. 1D and E). To further select the key gene, Patu-8988 cells were subcutaneously inoculated in nude mice. After tumor formation, mice were treated with phosphate-buffered saline (PBS) plus GEM or hemin plus GEM. By measuring the tumor sizes, we found that hemin did not significantly enhance the effect of GEM but instead promoted tumor growth (Fig. 1F and G). Additionally, we performed RNA sequencing (Fig. S1) on xenograft tissue to search the differentially expressed genes between the two groups, and 37 up-regulated genes along with 11 down-regulated genes were finally identified (Fig. 1H; Fig. S2A). By considering the overlapping significantly up-regulated genes in ferroptosis inducer-resistant cells and tissue RNA sequencing, IRP1 was finally identified. To further confirm the role of IRP1 in drug resistance and iron tolerance of PCa *in vivo*, we performed immunohistochemistry staining and found that the level of IRP1 was significantly higher in drug-resistant tumor tissues of xenografts (Fig. S2B). To further verify the above results, we detected the expression of IRP1 in the six PCa cell lines. IRP1 showed significant overexpression in Patu-8988, PANC-1, and CFPAC1 cell lines at the protein level and mRNA level (Fig. 1I and J).

### IRP1 enhances the chemoresistance of PCa cells both *in vitro* and *in vivo*

**Figure 2:**
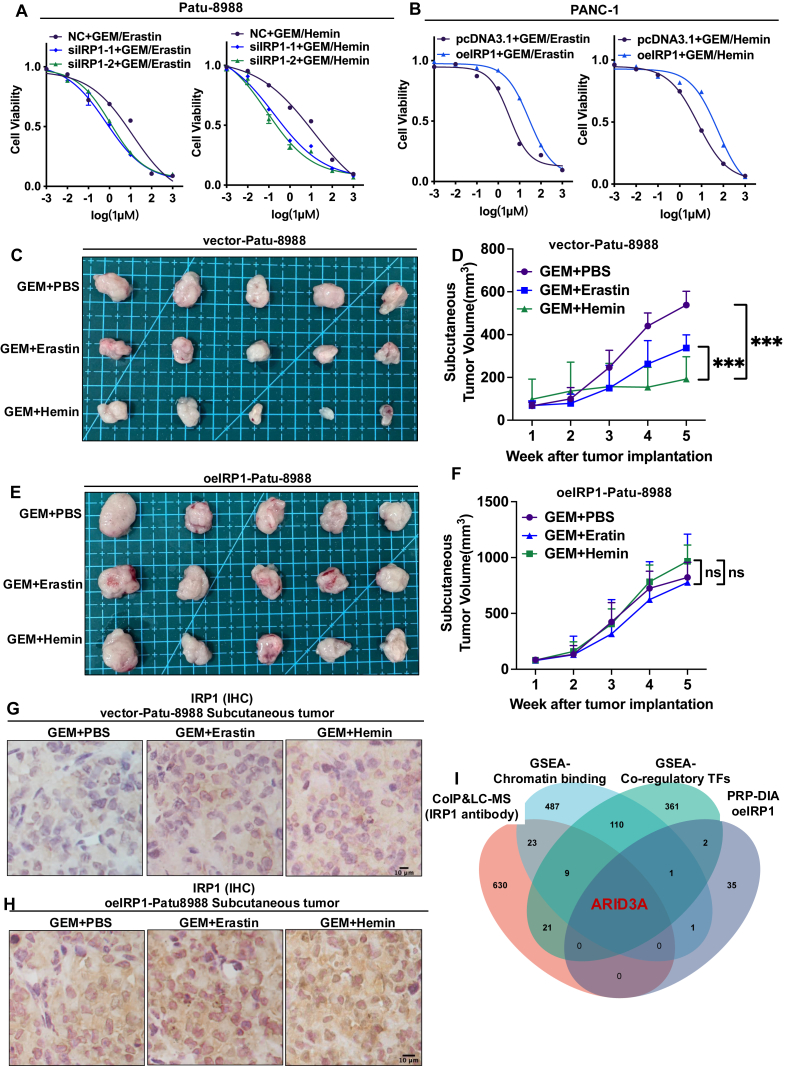
IRP1 promotes pancreatic cancer GEM-resistant *in vivo* and *in vitro.***(A)** IC50 curves of GEM plus erastin/hemin in IRP1-knockdown Patu-8988 cell line. **(B)** IC50 curves of GEM plus erastin/hemin in IRP1-overexpressing PANC-1 cell line. **(C)** Patu-8988 cells stably transfected with the empty vector were subcutaneously inoculated in nude mice. Afterward, the mice were subjected to injection with GEM plus PBS, GEM plus erastin, or GEM plus hemin. **(D)** Tumor growth curves of the three groups of nude mice (∗∗∗*P* < 0.001). **(E)** Patu-8988 cells stably transfected with the IRP1-overexpressing lentivirus were subcutaneously inoculated in nude mice. Afterward, the mice were subjected to injection with GEM plus PBS, GEM plus erastin, or GEM plus hemin. **(F)** Tumor growth curves of the three groups of nude mice (ns: *P* > 0.05). **(G, H)** The expression of IRP1 was detected in tumor tissue sections from the xenografts using immunohistochemistry. **(I)** The results of the intersection for co-immunoprecipitation and liquid chromatography-mass spectrometry against IRP1 antibody, gene set enrichment analysis (GSEA) databases (chromatin binding, co-regulatory transcription factors), and PRO-DIA (data-independent acquisition). GEM, gemcitabine; PBS, phosphate-buffered saline.

To explore the functional roles of IRP1 in PCa chemoresistance, we performed functional experiments to verify the effect of IRP1 on the malignant biological behavior of PCa cells. IRP1 was silenced in Patu8988 cells with siRNA and overexpressed in PANC-1 cells with an overexpression plasmid. The knockdown and overexpression of IRP1 were evaluated using Western blotting (Fig. S2C and S2D) and quantitative reverse transcription PCR (Fig. S2E and S2F). PCa cells were treated with GEM plus hemin or erastin. The cytotoxicity assay indicated that cells with higher IRP1 expression had higher IC50 values, while cells with lower IRP1 expression were more sensitive to drug treatment (Fig. 2A and B). Additionally, we established pancreatic xenograft tumors to further confirm the relationship between IRP1 and drug resistance (GEM and ferroptosis inducers) *in vivo*. Clear tumor formation was observed after 1.5 weeks of injection, and subsequently subjected to treatment with GEM plus PBS, GEM plus hemin, or GEM plus erastin. Compared with the control group, overexpression of IRP1 showed significant promoting effects on tumor growth in PCa. Furthermore, in the mice with IRP1 overexpression, the application of hemin or erastin did not improve the curative effect of GEM (Fig. 2C–F). Subsequently, we performed immunohistochemistry staining and found that the oeIRP1-treated GEM plus hemin group and GEM plus erastin group obtained higher staining scores of IRP1 among six groups (Fig. 2G and H). Taken together, our results suggest that overexpression of IRP1 renders PCa more resistant to GEM and ferroptosis inducers both *in vivo* and *in vitro*.

### ARID3A was identified as a crucial target of IRP1

**Figure 3:**
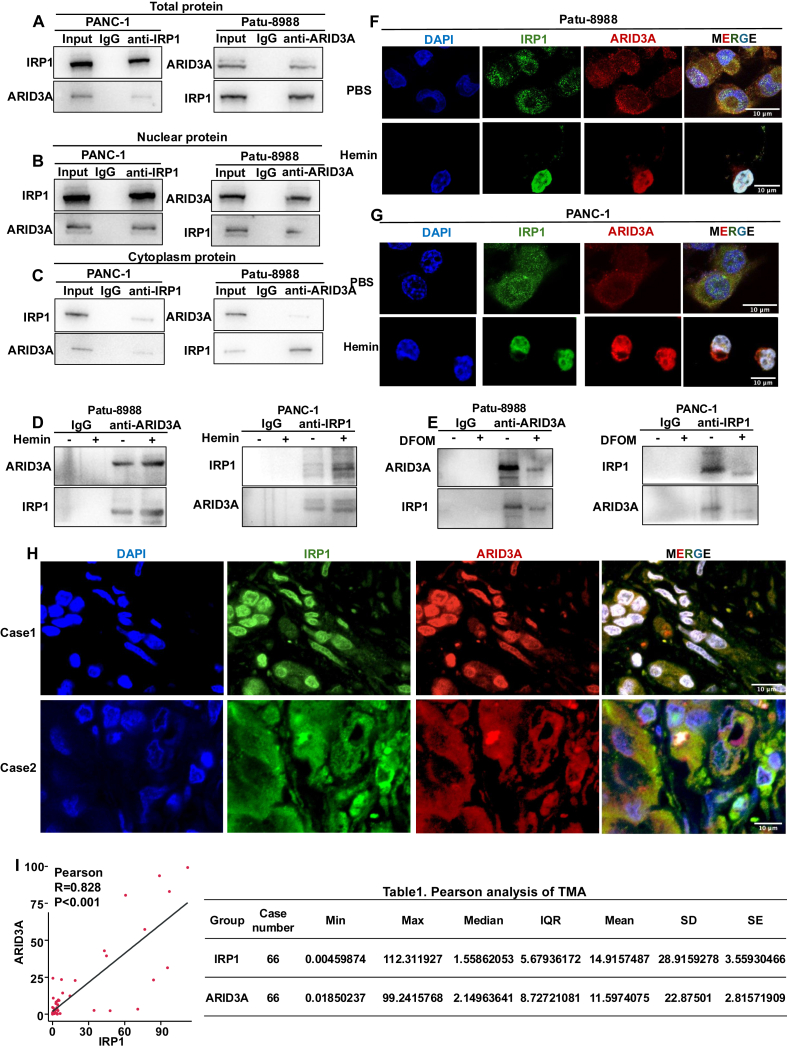
IRP1 transports ARID3A into the nucleus under the condition of iron overload. **(A)** Co-immunoprecipitation results of IRP1 and ARID3A using total protein in PANC-1 and Patu-8988 cells. **(B)** Co-immunoprecipitation results of IRP1 and ARID3A using nuclear protein in PANC-1 and Patu-8988 cells. **(C)** Co-immunoprecipitation results of IRP1 and ARID3A using cytoplasmic protein in PANC-1 and Patu-8988 cells. **(D)** Co-immunoprecipitation results of IRP1 and ARID3A using total protein in PANC-1 and Patu-8988 cells with or without hemin. **(E)** Co-immunoprecipitation results of IRP1 and ARID3A using total protein in PANC-1 and Patu-8988 cells with or without DFOM. **(F)** Cellular localization of IRP1 and ARID3A under normal or iron overload conditions in Patu-8988 cells. **(G)** Cellular localization of IRP1 and ARID3A under normal or iron overload conditions in PANC-1 cells. **(H)** Cellular localization of IRP1 and ARID3A in pancreatic cancer tissues. **(I)** Pearson correlation analysis of IRP1 and ARID3A nuclear localization in the tissue microarray (*r* = 0.828, *P* < 0.001). DFOM, desferroxamine.

To investigate the in-depth mechanism of action of IRP1 in PCa chemoresistance, we performed co-immunoprecipitation, mass spectrometry, and PRO-DIA proteome (IRP1-overexpressed) to identify the proteins that could interact with IRP1 (Fig. S3A). By considering the overlapping significantly up-regulated genes among liquid chromatography-mass spectrometry, proteome, and two GSEA datasets (GOMF_CHROMATIN_BINDING, GOMF_TRANSCRIPTION_COREGULATOR_ACTIVITY), ARID3A was found to be associated with chromatin binding and chemoresistance of PCa, and interacted directly with IRP1^18^ (Fig. 2I; Fig. S3B and S3C). Given that IRP1 is a crucial factor involved in iron homeostasis, we manipulated the iron concentration of PCa cells and performed co-immunoprecipitation assays. As the results shown, firstly, we found a significant interaction between IRP1 and ARID3A in total cellular proteins (Fig. 3A). Then, we extracted nuclear proteins and cytoplasmic proteins separately, and found that under the condition of iron overload, IRP1 and ARID3A interacted tightly in the nuclear fraction (Fig. 3B), while no obvious bands were found in the cytoplasmic proteins, even after hemin treatment (Fig. 3C). To further demonstrate that iron overload promoted the binding of IRP1 to ARID3A, we pre-treated PCa cells with hemin and desferroxamine, and then extracted total protein for co-immunoprecipitation validation. The results showed that hemin treatment significantly increased the binding of ARID3A and IRP1, while desferroxamine treatment significantly inhibited their binding (Fig. 3D and E). Immunofluorescence staining also showed that ARID3A co-localized with IRP1 under the iron overload condition in PCa cells, while no significant co-localization was observed in the control group (Fig. 3F and G). In addition, we conducted immunofluorescence staining in a tissue microarray of PCa patients, and the results showed that IPR1 and ARID3A had different degrees of co-localization (Fig. 3H). By analyzing the localization of IRP1 and ARID3A in the nucleus and cytoplasm, we found that IRP1 and ARID3A were positively correlated in the nuclear localization in PCa tissues (Fig. 3I and Table 1).

**Table 1:** IRP1 and ARID3A were positively correlated in the nuclear localization in PCa tissues.

Group	Case number	Min	Max	Median	IQR	Mean	SD	SE
**IRP1**	66	0.00459874	112.311927	1.55862053	5.67936172	14.9157487	28.9159278	3.55930466
**ARID3A**	66	0.01850237	99.2415768	2.14963641	8.72721081	11.5974075	22.87501	2.81571909

**Figure 4:**
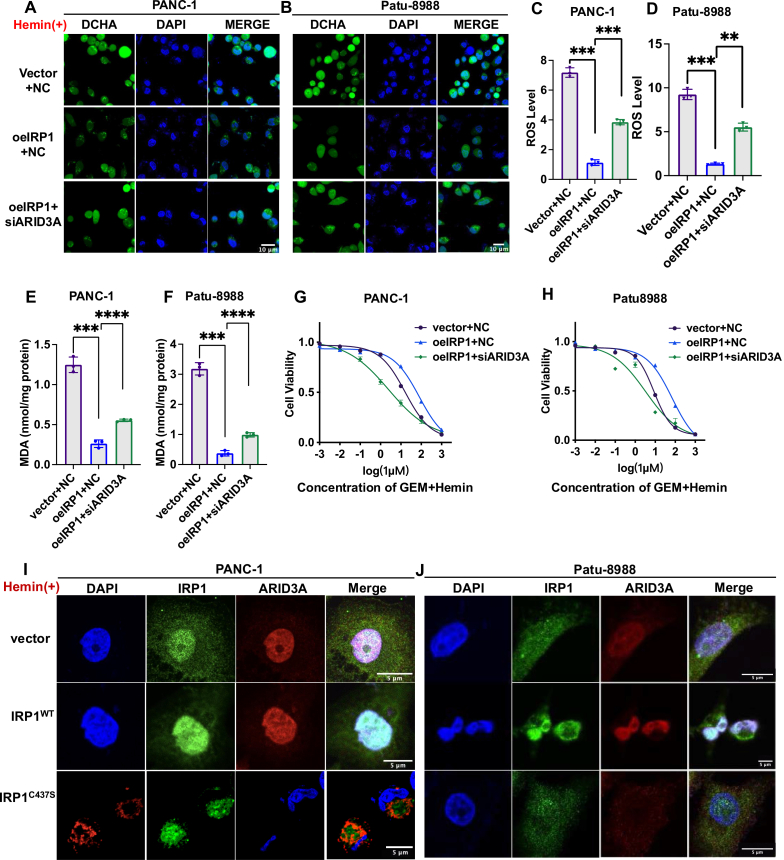
holo-IRP1/ARID3A axis promotes pancreatic cancer cells resistant to ferroptosis and chemotherapy. **(A)** DCFH-DA staining (green) was used to detect ROS generation in PANC-1 cells with empty vector, overexpression plasmids targeting IRP1, or overexpression plasmids targeting IRP1 and RNAi targeting ARID3A transfected. The nuclei were stained blue with Hochest33342. **(B)** DCFH-DA staining (green) was used to detect ROS generation in Patu-8988 cells with empty vector, overexpression plasmids targeting IRP1, or overexpression plasmids targeting IRP1 and RNAi targeting ARID3A transfected. The nuclei were stained blue with Hochest33342. **(C)** The oxygen consumption rates of PANC-1 cells were showed as a bar chart (∗∗∗*P* < 0.001). **(D)** The oxygen consumption rates of Patu-8988 cells were shown as a bar chart (∗∗∗*P* < 0.001, ∗∗*P* < 0.01). **(E)** MDA levels were detected in PANC-1 cells with empty vector, overexpression plasmids targeting IRP1, or overexpression plasmids targeting IRP1 and RNAi targeting ARID3A transfected (∗∗∗*P* < 0.001, ∗∗∗∗*P* < 0.0001). **(F)** MDA levels were detected in Patu-8988 cells with empty vector, overexpression plasmids targeting IRP1, or overexpression plasmids targeting IRP1 and RNAi targeting ARID3A transfected (∗∗∗*P* < 0.001, ∗∗∗∗*P* < 0.0001). **(G)** IC50 curves of GEM plus hemin in PANC-1 cells with empty vector, overexpression plasmids targeting IRP1, or overexpression plasmids targeting IRP1 and RNAi targeting ARID3A transfected. **(H)** IC50 curves of GEM plus hemin in Patu-8988 cells with empty vector, overexpression plasmids targeting IRP1, or overexpression plasmids targeting IRP1 and RNAi targeting ARID3A transfected. **(I)** Cellular localization of IRP1 and ARID3A in PANC-1 cells transfected with vector, IRP1^WT^, or IRP1^C437S^ lentivirus. **(J)** Cellular localization of IRP1 and ARID3A in Patu-8988 cells transfected with vector, IRP1^WT^, or IRP1^C437S^ lentivirus. MDA, malondialdehyde; GEM, gemcitabine; DCFH-DA, 2′,7′-dichlorofluorescin diacetate; ROS, reactive oxygen species.

Then, we detected the relative levels of lipid ROS and malondialdehyde (MDA). The results showed that overexpression of IRP1 significantly inhibited lipid ROS and MDA levels, and ARID3A silencing led PCa cells to high ROS and MDA levels (Fig. 4A–F). IC50 value also revealed that overexpression of IRP1 caused PCa cells to be more resistant, and knocking down ARID3A alleviated this effect (Fig. 4G and H). In addition, to comprehensively evaluate the effect of IRP1/RID3A on PCa chemo-resistance, we also examined cell apoptosis and cell cycle. The results showed that overexpression of IRP1 significantly reduced cell apoptosis rate, and ARID3A silencing led to PCa cell apoptosis (Fig. S4A and S4B). Overexpression of IRP1 was associated with a reduced population in the G1-phase and an enlarged population in the S-phase of the cell cycle, and ARID3A silencing expanded the G1-phase population and reduced the S-phase population (Fig. S4C and S4D).

### Subcellular localization of holo-IRP1 and ARID3A

Next, we further explored the mechanism of nuclear translocation of IRP1 and ARID3A under the condition of iron overload. In a previous study, a single point mutation was found in drosophila (IRP1^C450S^) that prevents the Fe–S cluster from binding to IRP1, further leading to the inability of IRP1 to enter the nucleus.^11^ Inspired by this finding, we compared the IRP1 sequence of drosophila with the human IRP1 sequence, and found that this mutant locus was located in the human IRP1^C437S^. Herein, we constructed the IRP1^C437S^ mutant and wild-type lenti-viruses separately and transfected them into human PCa cell lines. In IRP1 wild-type PCa cells, IRP1 and ARID3A were co-localized in the nucleus, while PCa cells with IRP1^C437s^ mutation had less nuclear translocation of IRP1, and no co-localization was observed (Fig. 4I and J).

**Figure 5:**
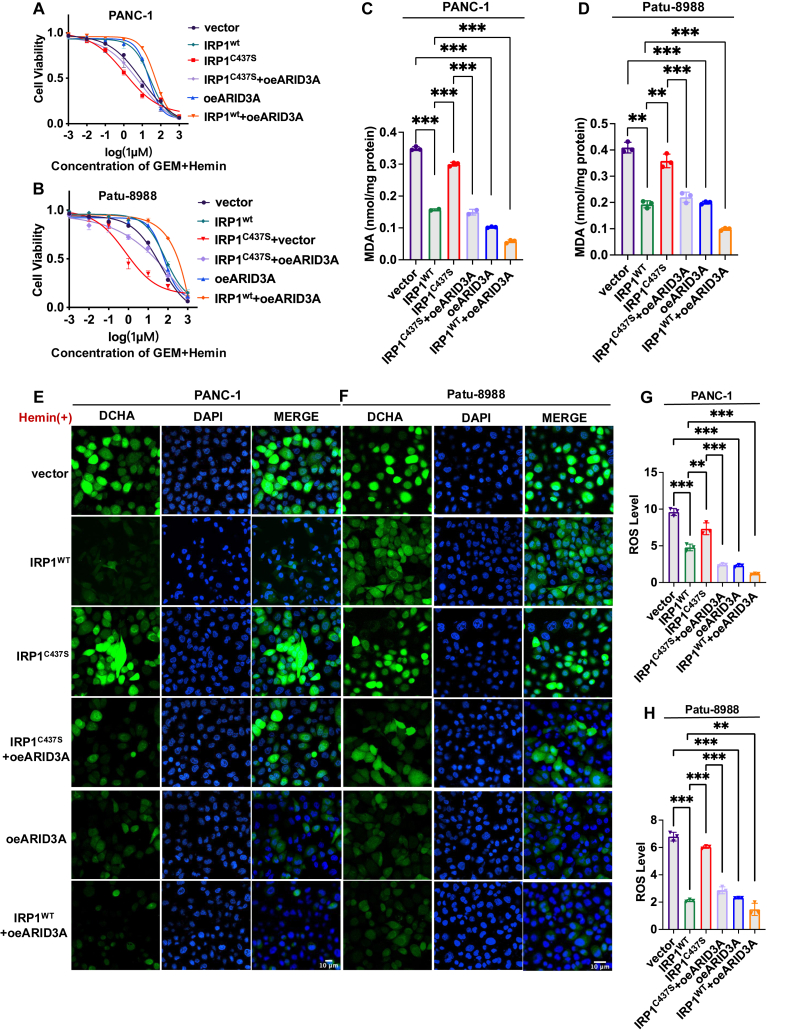
IRP1^C437S^ weakens the function of holo-IRP1 in pancreatic cancer cells. **(A)** IC50 curves of GEM plus hemin in PANC-1 cells transfected with vector, IRP1^WT^, IRP1^C437S^, IRP1^C437S^ plus ARID3A overexpression plasmid, ARID3A overexpression plasmid, and IRP1^WT^ plus ARID3A overexpression plasmid. **(B)** IC50 curves of GEM plus hemin in Patu-8988 cells transfected with vector, IRP1^WT^, IRP1^C437S^, IRP1^C437S^ plus ARID3A overexpression plasmid, ARID3A overexpression plasmid, and IRP1^WT^ plus ARID3A overexpression plasmid. **(C)** MDA levels of PANC-1 cells transfected with vector, IRP1^WT^, IRP1^C437S^, IRP1^C437S^ plus ARID3A overexpression plasmid, ARID3A overexpression plasmid, and IRP1^WT^ plus ARID3A overexpression plasmid (∗∗∗*P* < 0.001). **(D)** MDA levels of Patu-8988 cells transfected with vector, IRP1^WT^, IRP1^C437S^, IRP1^C437S^ plus ARID3A overexpression plasmid, ARID3A overexpression plasmid, and IRP1^WT^ plus ARID3A overexpression plasmid (∗∗*P* < 0.01, ∗∗∗*P* < 0.001). **(E)** DCFH-DA staining (green) was used to detect ROS generation in PANC-1 cells transfected with vector, IRP1^WT^, IRP1^C437S^, IRP1^C437S^ plus ARID3A overexpression plasmid, ARID3A overexpression plasmid, and IRP1^WT^ plus ARID3A overexpression plasmid. **(F)** DCFH-DA staining (green) was used to detect ROS generation in Patu-8988 cells transfected with vector, IRP1^WT^, IRP1^C437S^, IRP1^C437S^ plus ARID3A overexpression plasmid, ARID3A overexpression plasmid, and IRP1^WT^ plus ARID3A overexpression plasmid. **(G)** The oxygen consumption rates of PANC-1 cells were shown as a bar chart (∗∗*P* < 0.01, ∗∗∗*P* < 0.001). **(H)** The oxygen consumption rates of Patu-8988 cells were shown as a bar chart (∗∗*P* < 0.01, ∗∗∗*P* < 0.001). MDA, malondialdehyde; GEM, gemcitabine; DCFH-DA, 2′,7′-dichlorofluorescin diacetate; ROS, reactive oxygen species.

To further investigate whether IRP1^C437S^ mutation would affect the function of IRP1 in PCa cells, cell viability was detected in IRP1^C437S^ cells and IRP1^WT^ cells. The results showed that compared with IRP1^WT^ cells, cells with IRP1^C437S^ showed a significant decrease in resistance to GEM plus hemin. Overexpression of ARID3A in IRP1^C437S^ cells enhanced cell drug resistance. Overexpression of ARID3A in IRP1^WT^ cells further enhanced cell resistance and was more resistant than overexpression of ARID3A or IRP1 alone (Fig. 5A and B). Moreover, ROS level and MDA level were also detected. Compared with wild-type IRP1, C437S mutation led to an increase in ROS level and MDA level. Based on IRP1^C437S^, overexpression of ARID3A in IRP1^C437S^ cells led to a relative decrease in ROS level and MDA level. Overexpression of ARID3A in IRP1^WT^ cells had a more pronounced attenuation of cellular ROS levels and ferroptosis (Fig. 5C–H).

### IRP1 and ARID3A orchestrate transcription of CYGB in PCa cells

**Figure 6:**
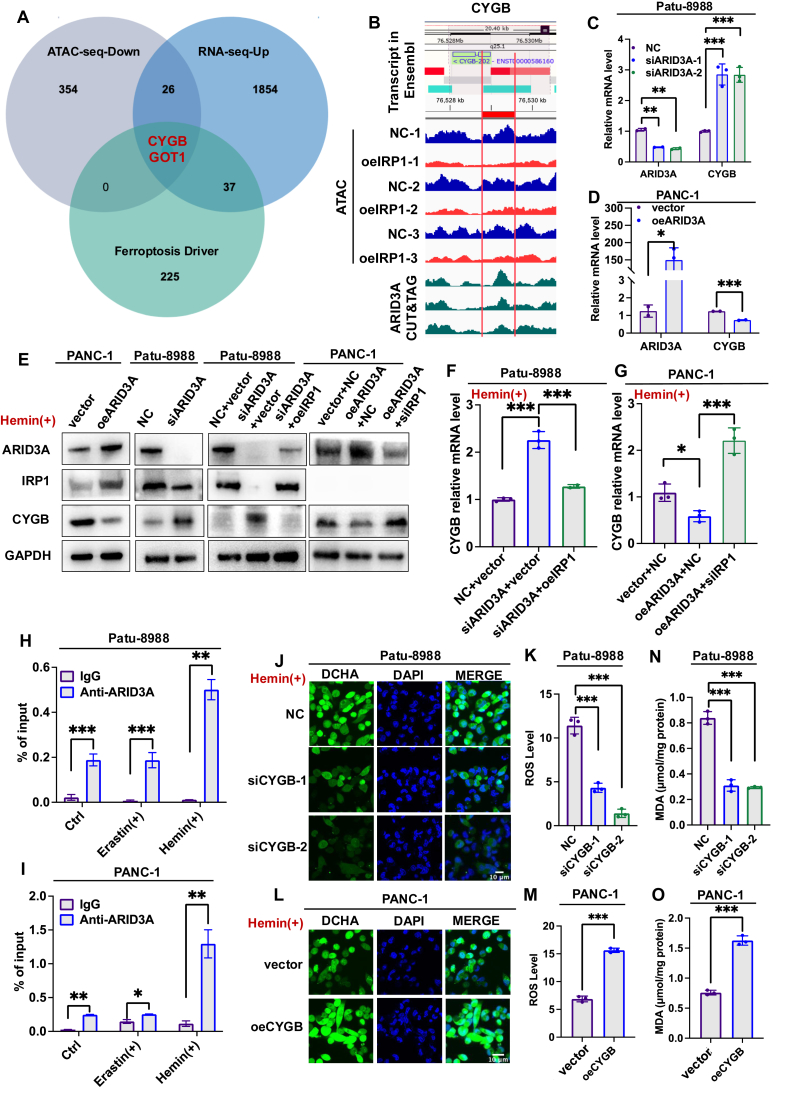
IRP1 and ARID3A reduce the chromatin accessibility of the CYGB promoter region and inhibit CYGB expression. **(A)** The intersection of down-regulated peaks in ATAC sequencing, up-regulated genes in RNA sequencing, and the Ferroptosis driver database. **(B)** The binding peak of ARID3A and chromatin accessibility regulated by IRP1 in the promoter region of CYGB were visualized by an integrative genomics viewer (IGV). **(C)** CYGB mRNA expression level in ARID3A-knockdown Patu-8988 cells was detected by quantitative reverse transcription PCR (∗∗*P* < 0.01, ∗∗∗*P* < 0.001). **(D)** CYGB mRNA expression level in ARID3A-overexpressing PANC-1 cells was detected by quantitative reverse transcription PCR (∗*P* < 0.05, ∗∗∗*P* < 0.001). **(E)** ARID3A, IRP1, and CYGB protein expression levels were detected in PANC-1 and Patu-8898 cells by Western blotting to verify the relationship between ARID3A, IRP1, and CYGB under the condition of iron overload. **(F)** CYGB expression level was detected in Patu-8898 cells by Western blotting to verify the relationship between ARID3A, IRP1, and CYGB under the condition of iron overload (∗∗∗*P* < 0.001). **(G)** CYGB expression level was detected in PANC-1 cells by Western blotting to verify the relationship between ARID3A, IRP1, and CYGB under the condition of iron overload (∗*P* < 0.05, ∗∗∗*P* < 0.001). **(H, I)** Chromatin immunoprecipitation assay performed with ARID3A antibody followed by detection of CYGB promoter through quantitative reverse transcription PCR in Patu-8988 and PANC-1 cells (normal condition, erastin added, hemin added; ∗*P* < 0.05, ∗∗*P* < 0.01, ∗∗∗*P* < 0.001). **(J)** DCFH-DA staining (green) was used to detect ROS generation in Patu-8988 cells transfected with control, siRNAs targeting CYGB. **(K)** The oxygen consumption rates of Patu-8988 cells were shown as a bar chart (∗∗∗*P* < 0.001). **(L)** DCFH-DA staining (green) was used to detect ROS generation in PANC-1 cells transfected with the vector, overexpression plasmid targeting CYGB. **(M)** The oxygen consumption rates of PANC-1 cells were shown as a bar chart (∗∗∗*P* < 0.001). **(N)** MDA levels were detected in Patu-8988 cells transfected with control, siRNAs targeting CYGB (∗∗∗*P* < 0.001). **(O)** MDA levels were detected in PANC-1 cells transfected with the vector, overexpression plasmid targeting CYGB (∗∗∗*P* < 0.001). MDA, malondialdehyde; DCFH-DA, 2′,7′-dichlorofluorescin diacetate; ROS, reactive oxygen species.

Given that previous research has shown that IRP1 inhibits the expression of iron-related genes after entering the nucleus,^19^ further investigations were conducted to explore how IRP1 and ARID3A inhibited ferroptosis by affecting iron-related genes on the epigenetic level. We used genome-wide ATAC sequencing (control and IRP1-overexpressed PANC-1 cells) (Fig. S5), CUT&Tag using an antibody against ARID3A (IRP1-overexpressed PANC-1 cells), and RNA sequencing (control and siARID3A) analysis to screen target genes that are regulated by IRP1 and ARID3A. Totally 382 down-regulated peaks were selected from ATAC sequencing (log_2_fold change < −0.5, annotation as promoter, *P* < 0.05), and 1919 up-regulated genes were selected from RNA sequencing (log_2_fold change > 0.5, *P* < 0.05). We took the intersection of the two gene sets, and 28 genes were found. To further screen out genes related to ferroptosis, we intersected the above 28 genes with 370 ferroptosis drivers from FerrDb V2 database (http://www.zhounan.org/ferrdb/current/), GOT1 and CYGB were identified for further investigation (Fig. 6A). We then analyzed the ARID3A CUT&Tag sequencing of the enhanced binding sites, and found that ARID3A binding was significantly enhanced in the CYGB promoter region (Fig. 6B). Therefore, CYGB might be the key gene that is regulated by ARID3A and IRP1 in the regulation of GEM resistance of PCa cells.

Then, we validated the regulation of IRP1 and ARID3A on CYGB expression at the protein and mRNA levels. Knocking down ARID3A led to high expression of CYGB, while overexpression of ARID3A inhibited CYGB expression (Fig. 6C and D). In addition, overexpressing IRP1 based on ARID3A knockdown led to decreased CYGB expression, while conversely, knocking down IRP1 based on ARID3A overexpression led to increased CYGB expression (Fig. 6E and F). To further confirm the role of IRP1 and ARID3A in CYGB expression, we performed chromatin immunoprecipitation assays in the two cell lines. As expected, the ARID3A antibody, rather than the IgG antibody, pulled down the promoter region of CYGB. In addition, we treated the cells with hemin and erastin, and it was found that hemin treatment further enhanced the binding between ARID3A and CYGB promoter fragments compared with control and erastin (Fig. 6H and I). To further determine whether such binding of ARID3A to CYGB promoter regions was functional, both wild-type and mutated CYGB promoter fragments were cloned into a luciferase reporter vector, which was transfected into PCa cells. Luciferase reporter assay results showed that up-regulation of ARID3A significantly increased relative luciferase activity compared with the wild-type CYGB promoter, while the fluorescence intensity of the mutated CYGB promoter group had no significant difference (Fig. S6). These results suggest that elevated iron level facilitates the binding of ARID3A to the promoter region of CYGB in an IRP1-dependent manner. After binding to the CYGB promoter, IRP1/ARID3A decreased the chromatin accessibility of this region, thereby inhibiting the transcription of CYGB.

### CYGB enhances ROS level and increases sensitivity to ferroptosis of PCa cells

As a regulator of ROS, CYGB plays a critical role in oxygen homeostasis and acts as a tumor suppressor.^16^^,^^20^ To further identify the role of CYGB in PCa cells, we transfected siRNA of CYGB in PANC-1 and overexpressed plasmids in Patu-8988 (Fig. S7A and S7B). ROS levels were measured in the above-treated cells. Knocking down CYGB led to a weakened ROS level, and overexpressing CYGB led to an obviously elevated ROS level. Then, we proved that CYGB promoted the ferroptosis of PCa cells by measuring the MDA level. Overexpression of CYGB promoted ferroptosis and MDA level of PCa cells, whereas knockdown of CYGB inhibited ferroptosis (Fig. 6J–O).

### The promoter region of CYGB is crucial for its functionality

**Figure 7:**
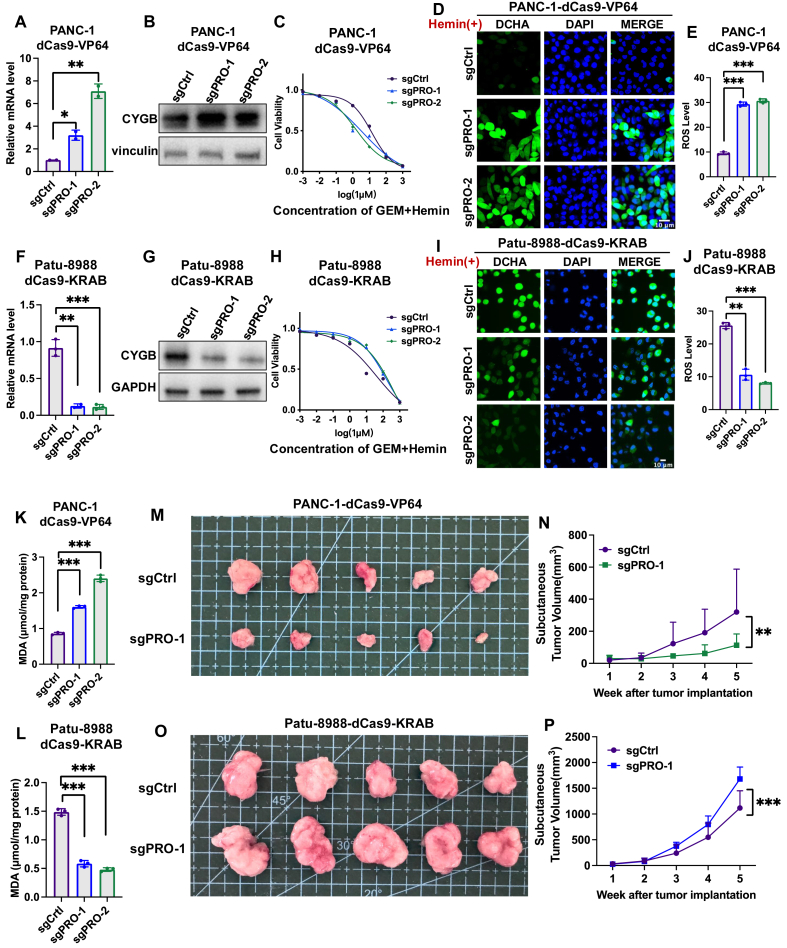
The promoter region of CYGB is crucial for its functionality. **(A)** CYGB mRNA expression level was detected by quantitative reverse transcription PCR in PANC-1 cells (stable expression of dCas9-VP64) transfected with control or sgRNAs targeting the CYGB promoter (∗*P* < 0.05, ∗∗*P* < 0.01). **(B)** CYGB protein level was detected by Western blotting in PANC-1 cells (stable expression of dCas9-VP64) transfected with control or sgRNAs targeting the CYGB promoter. **(C)** IC50 curves of GEM plus hemin in PANC-1 cells (stable expression of dCas9-VP64) transfected with control or sgRNAs targeting the CYGB promoter. **(D, E)** ROS levels of PANC-1 cells (stable expression of dCas9-VP64) transfected with control or sgRNAs targeting the CYGB promoter (∗∗∗*P* < 0.001). **(F)** CYGB mRNA expression level was detected by quantitative reverse transcription PCR in Patu-8988 cells (stable expression of dCas9-KRAB) transfected with control or sgRNAs targeting the CYGB promoter (∗∗*P* < 0.01, ∗∗∗*P* < 0.001). **(G)** CYGB protein level was detected by Western blotting in Patu-8988 cells (stable expression of dCas9-KRAB) transfected with control or sgRNAs targeting the CYGB promoter. **(H)** IC50 curves of GEM plus hemin in Patu-8988 cells (stable expression of dCas9-KRAB) transfected with control or sgRNAs targeting the CYGB promoter. **(I, J)** ROS levels of Patu-8988 cells (stable expression of dCas9-KRAB) transfected with control or sgRNAs targeting the CYGB promoter (∗∗*P* < 0.01, ∗∗∗*P* < 0.001). **(K)** MDA levels of PANC-1 cells (stable expression of dCas9-VP64) transfected with control or sgRNAs targeting the CYGB promoter (∗∗∗*P* < 0.001). **(L)** MDA levels of Patu-8988 cells (stable expression of dCas9-KRAB) transfected with control or sgRNAs targeting the CYGB promoter (∗∗∗*P* < 0.001). **(M)** PANC-1 cells (stable expression of dCas9-VP64) transfected with control and sgRNA targeting the CYGB promoter were subcutaneously inoculated in nude mice. **(N)** Tumor growth curves of the three groups of nude mice (∗∗*P* < 0.01). **(O)** Patu-8988 cells (stable expression of dCas9-KRAB) transfected with control and sgRNA targeting the CYGB promoter were subcutaneously inoculated in nude mice. **(P)** Tumor growth curves of the three groups of nude mice (∗∗∗*P* < 0.001). MDA, malondialdehyde; GEM, gemcitabine; ROS, reactive oxygen species.

To further confirm the function of the candidate promoter of CYGB, we applied the CRISPRa/i system to activate and silence the promoter region of CYGB. The sgRNAs targeting the CYGB promoter were cloned into the pX330a dCas9-KRAB vector and pX330a dCas9-VP64 vector, and then were transfected into the PCa cells (Fig. S7C). We found that the activation of the promoter region significantly enhanced the expression of CYGB (Fig. 7A and B). Cell viability, ROS level, and MDA level were detected to demonstrate the impact of the promoter region on CYGB function. PCa cells became more sensitive to GEM and hemin with the promoter region of CYGB activated (Fig. 7C). Activation of the promoter region led to increased ROS levels (Fig. 7D and E). In contrast, repression of the promoter region decreased the expression of CYGB (Fig. 7F and G). Repression of the promoter region led PCa cells to develop towards chemoresistance, and made them more resistant to ferroptosis (Fig. 7H–J). Furthermore, we detected MDA levels in cells with the CYGB promoter activated or suppressed, and found that activation of the CYGB promoter led to increased MDA levels, while inhibition of the promoter region led to decreased MDA levels (Fig. 7K and L).

Then, we established pancreatic xenograft tumors to validate the function of the CYGB promoter. The tumor growth rate and size were significantly decreased in the dCas9-VP64 group compared with the control group (Fig. 7M and N), while repression of the promoter region by the dCas9-KRAB vector caused increased tumor growth and size (Fig. 7O and P). In addition, immunohistochemistry staining against CYGB was performed in the tumor tissues of mice. Activation of the promoter region led to increased levels of CYGB expression in tumor tissues, and inhibition of the promoter region led to decreased levels of CYGB in tumor tissues (Fig. S7D and S7E).

**Figure 8:**
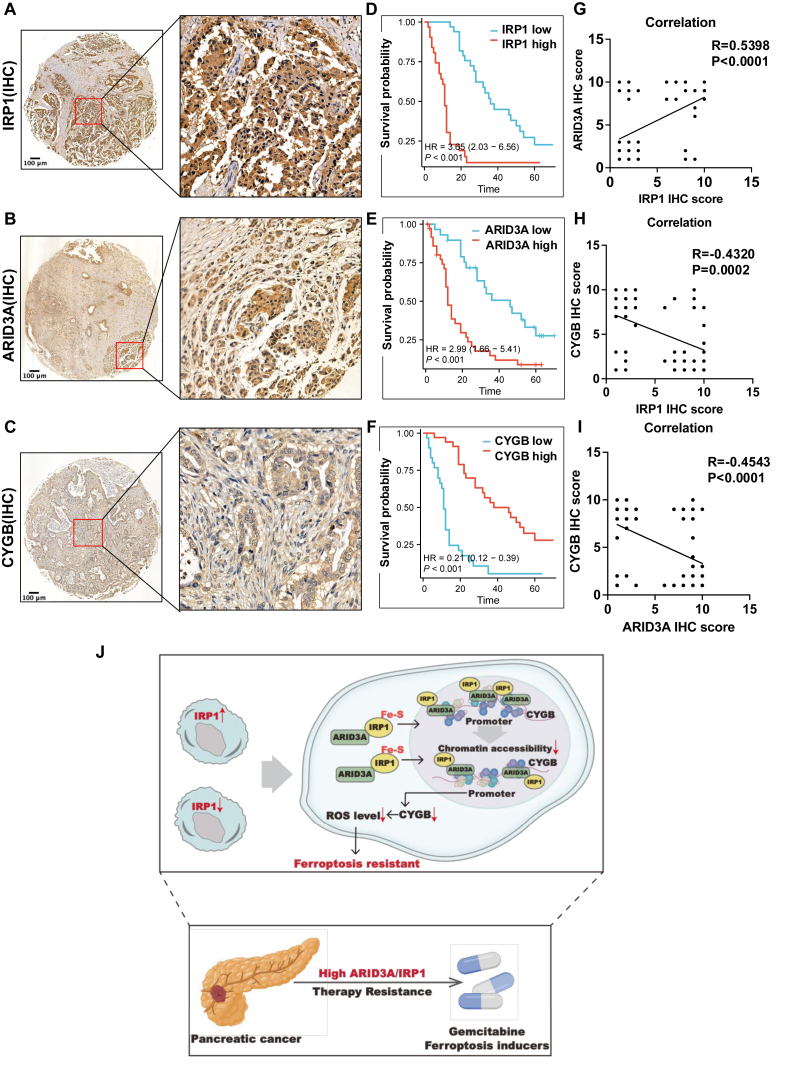
IRP1-ARID3A-CYGB axis plays an important role in PCa. **(A**–**C)** IRP1, ARID3A, and CYGB expression levels were detected by immunohistochemistry in PCa tissue microarrays. **(D)** High expression of IRP1 is associated with poor prognosis of PCa patients. **(E)** High expression of ARID3A is associated with poor prognosis of PCa patients. **(F)** Low expression of CYGB is associated with poor prognosis of PCa patients. **(G)** Correlation analysis of ARID3A and IRP1. **(H)** Correlation analysis of CYGB and IRP1. **(I)** Correlation analysis of CYGB and ARID3A. **(J)** Transcriptional inhibition of CYGB by IRP1/ARID3A leads to PCa cells resistant to ferroptosis and chemotherapy. PCa, pancreatic cancer.

To further clarify the clinical significance of ARID3A, IRP1, and CYGB in PCa, we detected the expression of these three in clinical samples. A tissue microarray containing PCa tissues of 66 cases was subjected to immunohistochemistry staining for ARID3A, IRP1, and CYGB (Fig. 8A–C). Importantly, compared with the low expression group, high ARID3A and IRP1 expression were related to shorter survival. In contrast, high CYGB was related to longer survival (Fig. 8D–F). ARID3A expression positively correlated with IRP1 (Fig. 8G). In addition, there was a negative relationship between IRP1 and CYGB and an obviously negative relationship between ARID3A and CYGB in the PCa tissues (Fig. 8H and I).

In summary, high expression of IRP1 and ARID3A leads to decreased chromatin accessibility at the CYGB promoter region, thereby contributing to the low expression of CYGB, which renders an unfavorable prognosis in PCa (Fig. 8J).

## Discussion

At present, the main treatment strategies for PCa include surgical resection and chemotherapy based on first-line drugs such as GEM. Effective chemotherapy can prolong the survival of patients with advanced diseases and transform unresectable or borderline resectable tumors into resectable tumors, increasing the likelihood of PCa patients undergoing surgical resection.^21^ Therefore, reducing the occurrence of chemotherapy resistance is a key aspect in cancer basic research. At present, first-line chemotherapy regimens for PCa mainly include combination chemotherapy regimens based on GEM (GEM combined with nab-paclitaxel), chemotherapy regimens based on 5-fluorouracil (FOLFIRINOX regimen), and platinum-based regimens applied to BRCA mutation patients (FOLFOX regimen).^22^ Many clinical trials have attempted to combine GEM with targeted drugs or alter the first-line drug combination regimen. However, satisfactory results have not been achieved. The mechanism of GEM resistance needs further exploration to discover combination targeted therapies that enhance GEM sensitivity.

Ferroptosis has been a research hotspot in cancer since the term was coined in 2012.^23^ Different from other types of cell death, the main characteristics of ferroptosis are redox state imbalance and increased ROS. Iron is a redox-active metal that can participate in the formation of free radicals and the spread of lipid peroxidation. Therefore, an increase in iron levels will increase the vulnerability to iron sagging. Various genes or proteins are involved in iron homeostasis, including their input, output, and storage.^24^ Numerous studies have shown that the ferroptosis pathway could affect the effectiveness of tumor treatment and even reverse chemotherapy resistance.^25^^,^^26^ According to the known mechanism of ferroptosis, three pathways are considered to reverse chemoresistance: GPX-regulated pathway, iron homeostasis, and lipid metabolism. Cisplatin is a chemotherapeutic drug for PCa, which is often used in combination with other drugs or radiotherapy. However, cisplatin has severe side effects and frequent drug resistance. Dihydroartemisinin is a ferroptosis inducer. Pre-clinical research in PCa found that it might be a safe and effective agent to reverse chemoresistance by inducing ferroptosis of PCa cells.^27^ In PCa, dihydroartemisinin can enhance the drug toxicity of cisplatin by destroying mitochondrial homeostasis, increasing mito-ROS and iron concentration, thereby promoting ferroptosis of PCa cells. In our study, we used two ferroptosis inducers, hemin and erastin, to detect the reactivity of different PCa cell lines. Erastin induces cytoplasmic ROS accumulation, while hemin leads to lipid instability by directly increasing intracellular iron concentration. By analyzing the differentially expressed genes between ferroptosis-sensitive and -tolerant cell lines, we conducted further exploration on IRP1, an iron regulatory protein. We found that the overexpression of IRP1 led to PCa cells being more tolerant to GEM plus ferroptosis inducers, while knocking down IRP1 promoted PCa cell death. In the section of introduction, we provided a detailed description of the physiological functions of IRP1, including its two forms (apo-IRP1 and holo-IRP1) at different intracellular iron concentrations, and its nuclear entry mechanism under iron overload. Some studies have elucidated the role of IRP1 in tumors. In non-small cell lung cancer, trabectedin increases iron and ROS levels by up-regulating transferrin receptor 1 (TFR1) and the hypoxia-inducible factor 1-alpha (HIF-1)/IRP1 axis, and leads to ferroptosis of non-small cell lung cancer cells.^28^ In hepatocellular carcinoma, enolase 1 (ENO1) inhibits mitochondrial ferritin-1 (Mfrn1) expression by recruiting CCR4-NOT transcription complex subunit 6 (CNOT6) to accelerate IRP1 decay in cancer cells, which in turn inhibits mitochondrial iron-induced ferroptosis.^29^ Thus, IPR1 plays an important role in tumor cells by regulating iron homeostasis. However, there is no research to clarify the regulation of IRP1 on chromatin accessibility in tumor cells, and there is no research about IRP1 in PCa.

In recent years, following histone modification and DNA modification, chromatin accessibility has become another key point in the epigenetic area. The degree of chromatin accessibility determines the activity of gene transcription. DNA sequences will be exposed during replication and transcription, and this exposed area is the chromatin open region, which can be bound by transcription factors and other regulatory elements.^30^ Combined with the functional description of IRP1 in previous studies, we found that in PCa, when the iron overload, IRP1 can translocate into the nucleus and regulate the expression of ferroptosis-related genes by affecting chromatin status. To find the transcription factors that bind to IRP1 in PCa cells, we performed co-immunoprecipitation and gel strip mass spectrometry identification. Based on the results of mass spectrometry, co-immunoprecipitation assays were conducted between IRP1 and ARID3A. These results revealed that ARID3A combined with IRP1 tightly under the condition of iron overload. As a transcription factor in the ARID family, ARID3A can regulate cancer cell proliferation, differentiation, and metastasis. In liver cancer, ARID3A and centrosomal protein 131 (CEP131) jointly act on the promoter region of lysine-specific demethylase 3A (KDM3A) and activate transcription, further up-regulating the expression of downstream embryonic stem cell characteristic genes through H3K9me2 demethylation.^31^ In PCa, inhibiting ARID3A alleviates the transcriptional inhibition of phosphatase and tensin homologue (PTEN), which leads to the reduction of GPX4 expression and promotes ferroptosis, and finally reverses GEM resistance of PCa.^18^ This study validated the phenotype at the organoid level, cellular level, and animal model level. In terms of molecular mechanism, the binding site of ARID3A on DNA was discovered through joint analysis of RNA sequencing and CUT&TAG sequencing, and its conclusion was repeatedly validated through experiments such as chromatin immunoprecipitation and quantitative PCR.

There are two opposing strategies for targeting tumors by regulating intracellular iron: one is iron depletion, including chelation; on the other hand, it is appropriate to utilize the toxic free radicals generated by iron overload. Desferoxamine is an existing clinically approved iron chelator for the treatment of iron overload, used to treat thalassemia and other iron overload patients.^32^^,^^33^ Some of the more successful anti-cancer iron chelators are amino thiourea derivatives, such as Dp44mT. Mechanism studies have shown that these three toothed chelating agents not only bind to intracellular iron, but also enable iron to undergo redox cycling, producing ROS that helps kill tumor cells.^34^ Dp44mT has entered clinical trials. Ferumoxynol is a clinically approved iron oxide nanoparticle used for treating iron deficiency. Ferumoxynol induced oxidative stress and reduced tumor burden in patient cells and mouse leukemia models.^35^ With the deepening of research on ferroptosis, iron-targeting drugs have emerged in tumor treatment. Cisplatin is a commonly used broad-spectrum anti-cancer drug. Ferroptosis may enhance the anti-cancer effect of the apoptosis inducer cisplatin, indicating that ferroptosis inducers can be used to enhance the efficacy of traditional anticancer drugs.^36^ BRD4 inhibitor JQ1 regulates the expression of genes related to ferritin phagocytosis and ferritin deposition (*e.g.*, GPX4, SCL3A2, SLC7A11) at the epigenetic level by inhibiting the expression of histone methyltransferase G9a or enhancing the expression of histone deacetylase sirtuin 1 (SIRT1). This suggests that the combination of JQ1 and ferroptosis inducers may become a new therapeutic approach.^37^

With the rapid development of bio-nanotechnology, various nano-formulations have been applied in clinical practice, such as doxorubicin liposomes (Doxil) and paclitaxel albumin nanoparticles (Abraxan).^38^^,^^39^ This provides a new approach for the *in vivo* delivery of ferroptosis inducers with poor water solubility, strong systemic toxicity, and low tumor delivery efficiency, laying the foundation for the development of new efficient and low-toxicity cancer therapeutic agents. Iron-containing nano metal organic frameworks (MOFs) contain a large amount of iron and porous structures, making them ideal iron donors and drug delivery systems. Tf-LipoMof@PL is a highly efficient ferroptosis/pyroptosis nano delivery system. Piperlongumine is an effective ferroptosis inducer loaded in MOFs coated with a DOPE pH-sensitive lipid layer with transferrin decoration. Iron concentration was modulated by MOFs and transferrin, and H_2_O_2_ was provided by piperlongumine. Fenton reaction and cellular ROS can be triggered by the nano-combination, thereby leading to the death of cancer cells.^40^ Pt-FMO is a manganese-deposited iron oxide nanoplatform loaded with cisplatin prodrug, which triggers intracellular cascade reactions, leading to the production of ROS and enhancing ferroptosis. Pt-FMO enables the tumor microenvironment to respond to the release of manganese, iron ions, and Pt-drugs. Manganese catalyzes a stronger Fenton reaction. Coupled with Pt-drugs that can promote the production of H_2_O_2_ in cancer cells, Pt-FMO is expected to significantly enhance the catalytic effect of the Fenton reaction, thereby benefiting the iron poisoning effect.^41^ However, there is no report that the related molecular drugs have achieved good results in the clinical treatment of PCa. This shows that inhibiting a certain iron metabolism-related gene alone is not enough to reverse the current status of drug resistance in PCa, so it is necessary to further combine the well-known target with regulating the iron homeostasis of cells to invent effective targeted drugs with multiple pathways.

In our study, we found that iron overload of PCa cells could change the morphology of IRP1 (holo-IRP1) and promote IRP1 to carry ARID3A into the nucleus. Considering that IRP1 could regulate chromatin stage and ARID3A is a transcription factor, we performed RNA sequencing, ATAC-sequencing, and CUT&TAG profiling. Through analyzing the results, CYGB was finally identified as the key downstream gene of IRP1/ARID3A, leading to drug resistance in PCa. Several studies have demonstrated that down-regulation of CYGB contributes to the progression of cancer. For instance, a study in liver cancer found that CYGB deficiency activated the oxidative stress pathway, thereby promoting liver fibrosis and cancer initiation.^42^ In colon cancer, overexpression of CYGB increases ROS accumulation and disrupts mitochondrial function, and suppresses cancer progression through the p53/YAP1 axis.^16^ However, a study demonstrated that CYGB inhibited cancer progression by scavenging ROS levels in PCa.^43^ We wonder how CYGB reverses drug resistance in PCa under iron overload. Hemin was added in PCa cells, and then, CYGB was overexpressed or knocked down. According to the results, CYGB stimulates ROS elevation and promotes ferroptosis, especially under the condition of iron overload. Compared with other current studies on ferroptosis, the innovation of our research is: i) The mechanism by which IRP1 promotes drug resistance in PCa cells was elucidated by its nuclear entry under the fluctuation of iron and its own regulatory function on iron metabolism. ii) Combining iron fluctuations, nuclear translocation of iron-metabolizing proteins, and transcription factor binding, a pathway by which IRP1/ARID3A jointly inhibits iron death and promotes drug resistance in PCa cells at the protein level and the epigenetic level was identified. iii) The combination of multi-targeted iron-related drugs with chemotherapeutic agents may improve the prognosis of PCa patients in future clinical translation.

In conclusion, IRP1 expression was significantly up-regulated under iron overload. The oncogenic role of IRP1 was identified using *in vitro* and *in vivo* functional assays. ARID3A was found directly interacting with IRP1 and binding to the promoter region of CYGB. Transcriptional inhibition of CYGB by IRP1/ARID3A led to PCa cells resistant to ferroptosis and chemotherapy. These findings indicate that targeting ferroptosis via iron concentration and IRP1/ARID3A sheds light on the development of novel therapeutic strategies for PCa.

## CRediT authorship contribution statement

**Zeru Li:** Writing – original draft, Conceptualization, Methodology, Visualization. **Cheng Qin:** Writing – original draft, Writing – review & editing, Conceptualization, Methodology. **Bangbo Zhao:** Visualization. **Tianyu Li:** Validation. **Yutong Zhao:** Visualization. **Lirui Huang:** Validation. **Haoyu Shi:** Writing – review & editing. **Yiping Xie:** Visualization. **Yutong Yan:** Visualization. **Xiangyu Zhang:** Validation. **Weibin Wang:** Conceptualization, Funding acquisition.

## Ethics declaration

The clinical PC specimens were collected with permission by the Institutional Research Ethics Committee of Peking Union Medical College Hospital, Beijing, China (I-23PJ2081). All the mouse protocols were reviewed and approved by the Ethics Committee of Peking Union Medical College Hospital (XHDW-2023-081).

## Data availability

The data used and/or analyzed during the current study are available from the corresponding author on reasonable request.

## Funding

This study received the support from the Beijing Natural Science Foundation (China) (No. 7232127 to W.W.B.), the National Natural Science Foundation of China (No. 82173074 to W.W.B.), the National High Level Hospital Clinical Research Funding (China) (No. 2022-PUMCH-B-004 to W.W.B.; 2022-PUMCH-D-001), the CAMS Innovation Fund for Medical Sciences (China) (No. 2021-I2M-1–002), the Nonprofit Central Research Institute Fund of Chinese Academy of Medical Sciences (No. 2018PT32014), the Postdoctoral Fellowship Program of China Postdoctoral Science Foundation (No. GZC20240146 to Q.C.), and the CAMS Innovation Fund for Medical Sciences (China) (No. 2023-I2M-2-002).

## Conflict of interests

The authors declared no competing interests.

## Appendix A Supplementary data

Multimedia component 2 Multimedia component 2

Figure S1 Workflow of RNA sequencing analysis. Figure S1

Figure S2 Volcano plot of differentially expressed genes of xenografts tumor tissue RNA sequencing, immunohistochemistry against IRP1 of xenograft tumor tissues, and transfection efficiency of siRNAs and plasmid of IRP1 and ARID3A. **(A)** Volcano plot of the differentially expressed genes between GEM plus PBS and GEM plus hemin of xenograft tissue RNA sequencing. **(B)** IRP1 immunohistochemistry staining of xenograft tumor tissues. **(C)** The transfection efficiency of siRNAs and overexpression plasmid targeting IRP1 was verified by western blotting. **(D)** The transfection efficiency of siRNAs and overexpression plasmid targeting ARID3A was verified by western blotting. **(E)** Transfection efficiency of siRNAs and overexpression plasmid targeting IRP1 was verified by quantitative reverse transcription PCR (∗∗∗*P* < 0.001). **(F)** Transfection efficiency of siRNAs and overexpression plasmid targeting ARID3A was verified by quantitative reverse transcription PCR (∗∗∗*P* < 0.001). GEM, gemcitabine; PBS, phosphate-buffered saline. Figure S2

Figure S3 Principal component analysis and gene ontology analysis of PRO-DIA proteome (IRP1-overexpressed and control). **(A)** Principal component analysis of the IRP1-overexpressed and control groups in the PRO-DIA proteome. **(B, C)** Gene ontology analysis of the differentially expressed genes between the IRP1-overexpressed and control groups in the PRO-DIA proteome. Figure S3

Figure S4 Regulation of holo-IRP1/ARID3A axis on pancreatic cancer cell apoptosis and cell cycle. **(A, B)** Apoptosis rate of pancreatic cancer cells with IRP1 overexpression and ARID3A silencing. **(C, D)** Cell cycle of pancreatic cancer cells with IRP1 overexpression and ARID3A silencing. Figure S4

Figure S5 Workflow of ATAC sequencing analysis. Figure S5

Figure S6 Luciferase analysis in PANC-1 and Patu-8988 cells. Relative luciferase intensity in PANC-1 and Patu-8988 cells transfected with corresponding designed plasmids. Figure S6

Figure S7 Transfection efficiency of siRNAs and plasmid of CYGB, design of CYGB promoter sgRNAs, and immunohistochemistry against CYGB. **(A)** The transfection efficiency of siRNAs and overexpression plasmid targeting CYGB was verified by western blotting. **(B)** Transfection efficiency of siRNAs and overexpression plasmid targeting CYGB was verified by quantitative reverse transcription PCR (∗∗∗*P* < 0.001). **(C)** Site pattern map of sgRNAs targeting the CYGB promoter region. **(D, E)** CYGB immunohistochemistry staining of xenograft tumor tissues. Figure S7
