# Supplementary material for: IRP1/ARID3A complex promotes pancreatic cancer chemoresistance by suppressing CYGB-related ferroptosis
Source: Genes Dis. 2025 Sep 24;13(5):101866. doi: 10.1016/j.gendis.2025.101866 (PMC13254595; doi:10.1016/j.gendis.2025.101866)
Supplement: Multimedia component 1 [file mmc1.docx]

| **Gene Names** | **Sequences** |
| --- | --- |
| IRP1-F | GTGATTGGCTACAGGCTGATGG |
| IRP1-R | GCGGAGGTGCTTGGTAATGG |
| ARID3A-F | CTCGTGGAGGTCATCAACAAGAAG |
| ARID3A-R | TATTGGGTCCGCAGGGTGAAG |
| CYGB-F | TACTTCAGCCAGTTCAAGCACATG |
| CYGB-R | CAGGTTCTCCACGACAGTGTTG |
| ACTB-F | TGGCACCCAGCACAATGAA |
| ACTB-R | CTAAGTCATAGTCCGCCTAGAAGCA |

**Table S1:The sequences of primers used in this article**
