# Supplementary material for: IRP1/ARID3A complex promotes pancreatic cancer chemoresistance by suppressing CYGB-related ferroptosis
Source: Genes Dis. 2025 Sep 24;13(5):101866. doi: 10.1016/j.gendis.2025.101866 (PMC13254595; doi:10.1016/j.gendis.2025.101866)
Supplement: Multimedia component 2 [file mmc2.docx]

**Table S2:** Data of antibodies used in this article

| **Antibody** | **WB** | **IHC** | **Co-IP** | **ChIP** | **Specificity** | **Company** |
| --- | --- | --- | --- | --- | --- | --- |
| IRP1 | 1:1000 | 1:200 | 1:15 | - | Mouse polyclonal | SantaCruz  (sc-166022) |
| ARID3A | 1:1000 | 1:150 | 1:20 | 1:30 | Rabbit polyclonal | Proteintech  (14068-1-AP) |
| CYGB | 1:1000 | 1:200 | - | - | Rabbit polyclonal | Proteintech  (13317-1-AP) |
| GAPDH | 1:2000 | - | - | - | Rabbit monoclonal | Proteintech  (10494-1-AP) |
| Vinculin | 1:2000 | - | - | - | Mouse  monoclonal | Proteintech  (26520-1-AP) |
