# Supplementary figures and images for: IRP1/ARID3A complex promotes pancreatic cancer chemoresistance by suppressing CYGB-related ferroptosis
Source: Genes Dis. 2025 Sep 24;13(5):101866. doi: 10.1016/j.gendis.2025.101866 (PMC13254595; doi:10.1016/j.gendis.2025.101866)

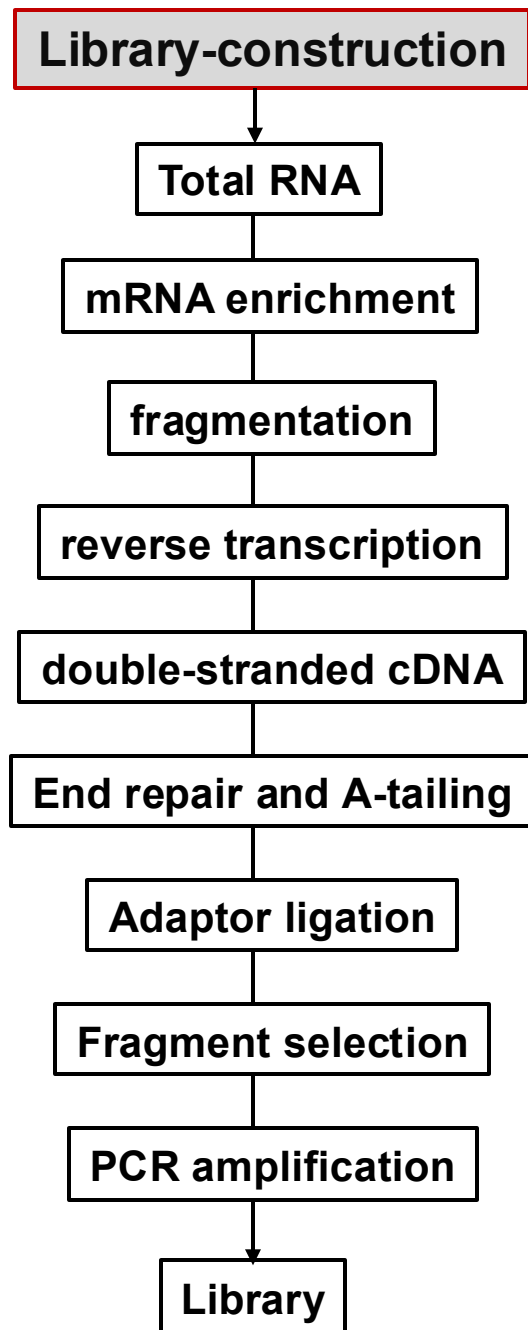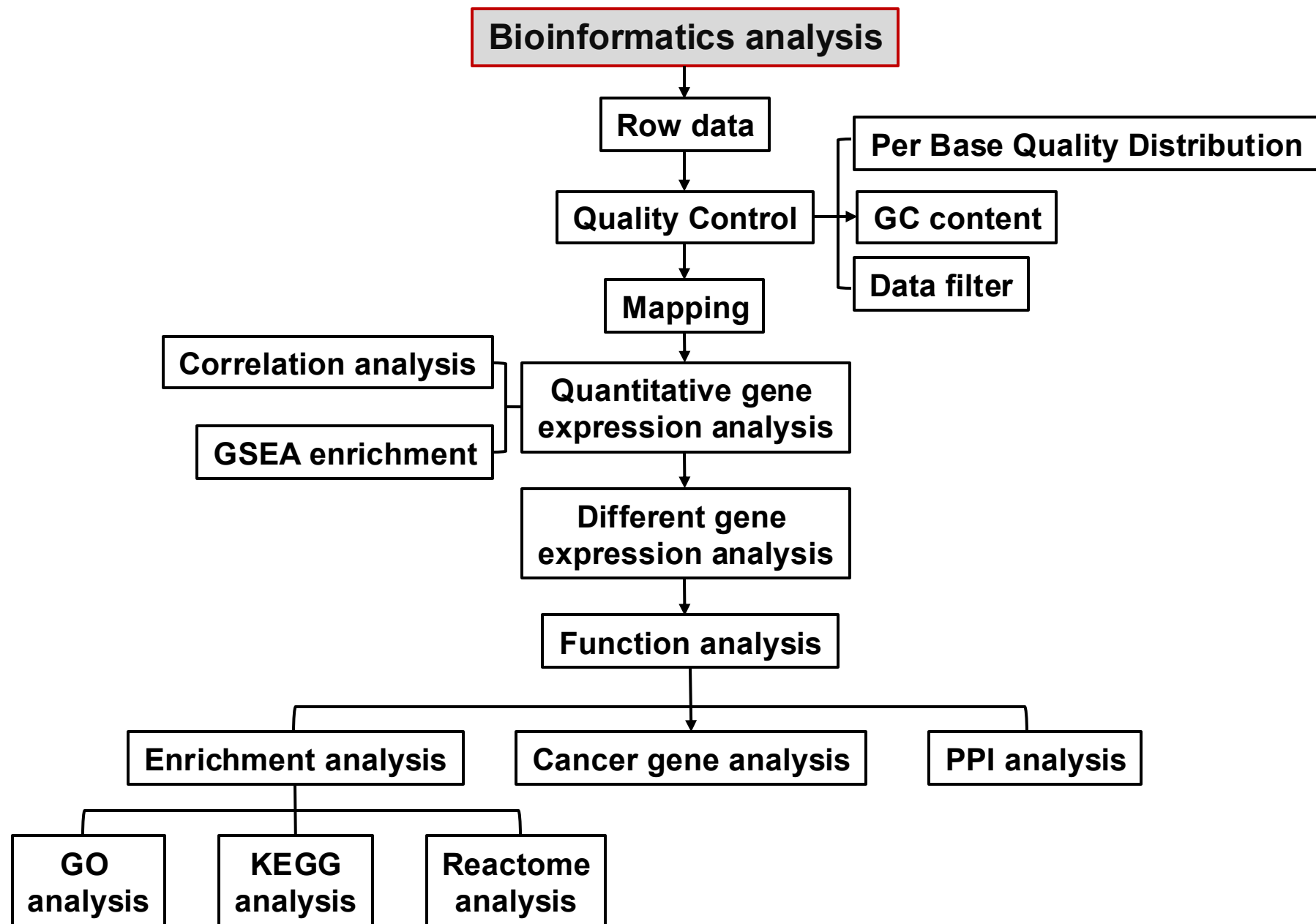

Supplement: Figure S1 — Workflow of RNA sequencing analysis. [file mmc3.pdf]

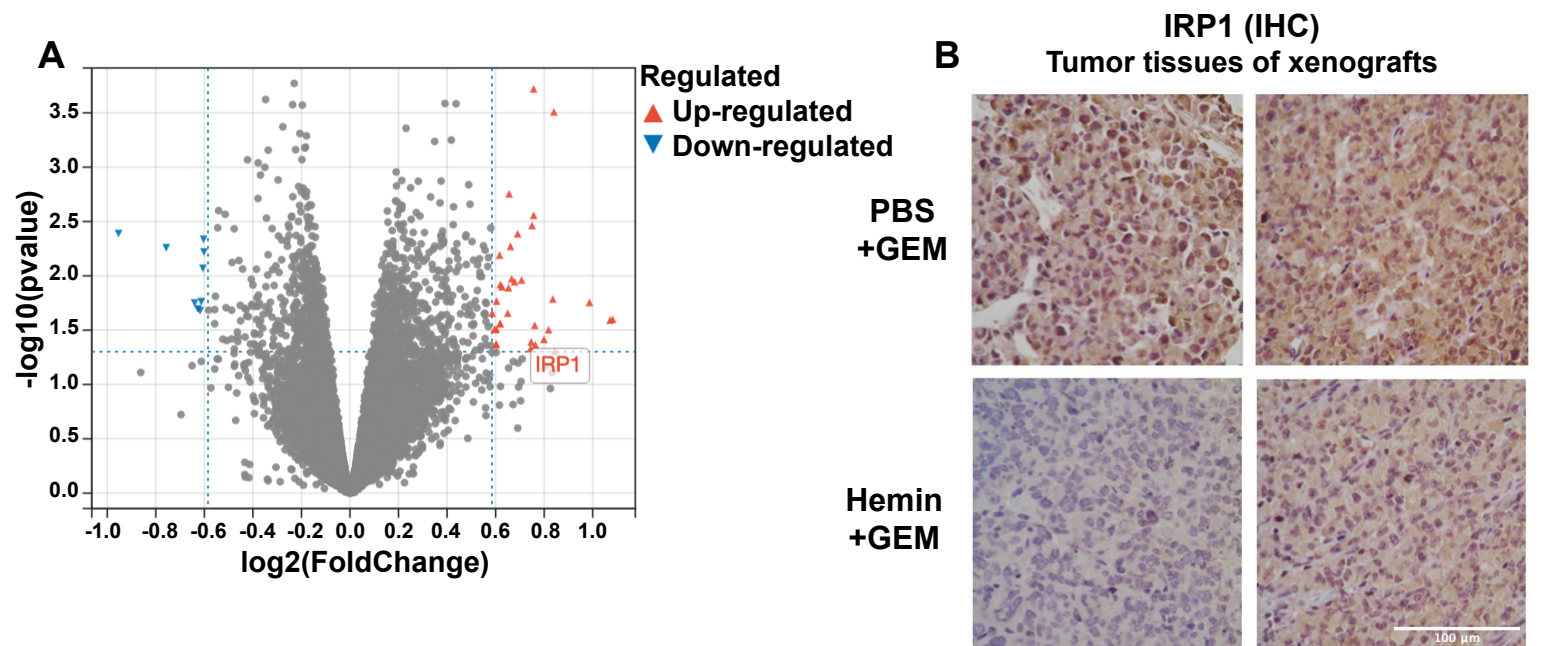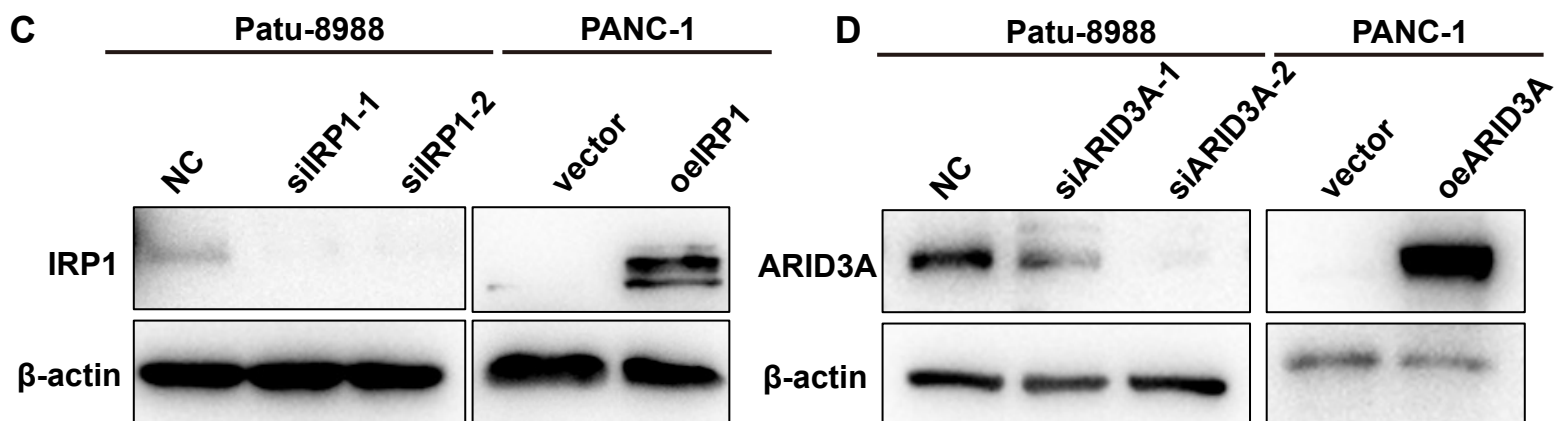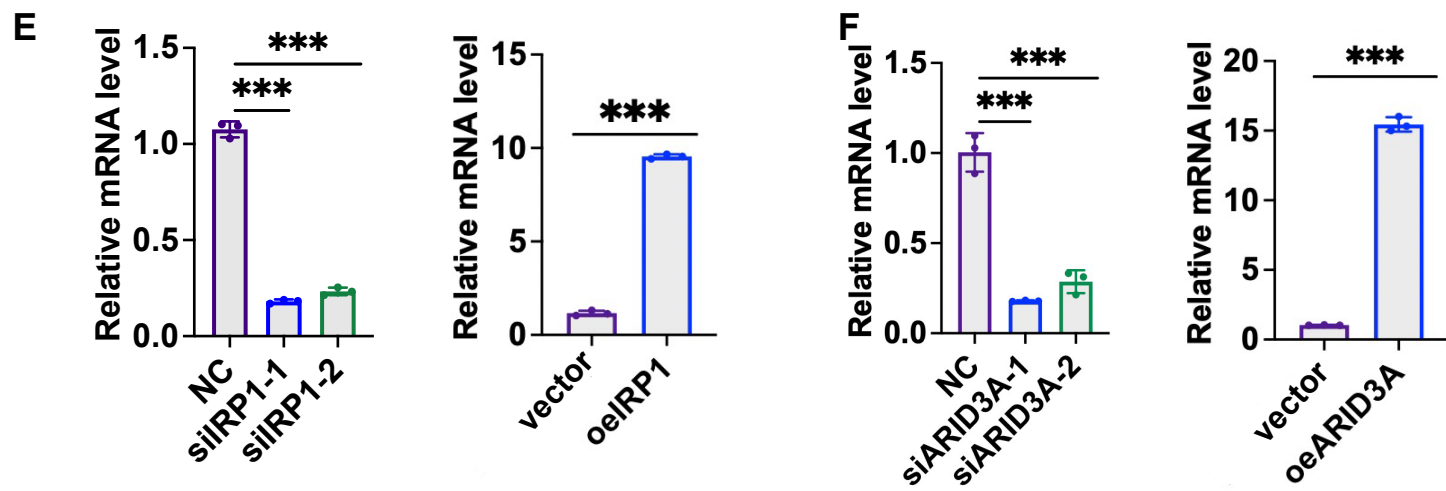

Supplement: Figure S2 — Volcano plot of differentially expressed genes of xenografts tumor tissue RNA sequencing, immunohistochemistry against IRP1 of xenograft tumor tissues, and transfection efficiency of siRNAs and plasmid of IRP1 and ARID3A. (A) Volcano plot of the differentially expressed genes between GEM plus PBS and GEM plus hemin of xenograft tissue RNA sequencing. (B) IRP1 immunohistochemistry staining of xenograft tumor tissues. (C) The transfection efficiency of siRNAs and overexpression plasmid targeting IRP1 was verified by western blotting. (D) The transfection efficiency of siRNAs and overexpression plasmid targeting ARID3A was verified by western blotting. (E) Transfection efficiency of siRNAs and overexpression plasmid targeting IRP1 was verified by quantitative reverse transcription PCR (∗∗∗P < 0.001). (F) Transfection efficiency of siRNAs and overexpression plasmid targeting ARID3A was verified by quantitative reverse transcription PCR (∗∗∗P < 0.001). GEM, gemcitabine; PBS, phosphate-buffered saline. [file mmc4.pdf]

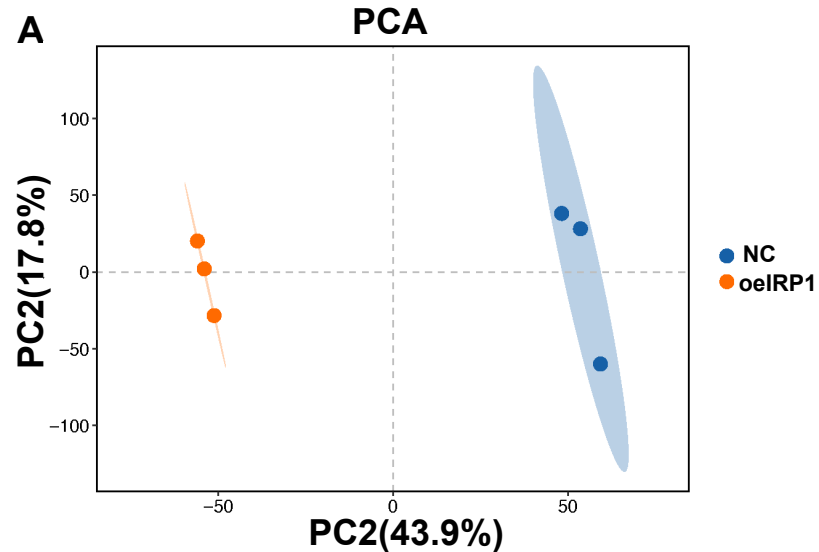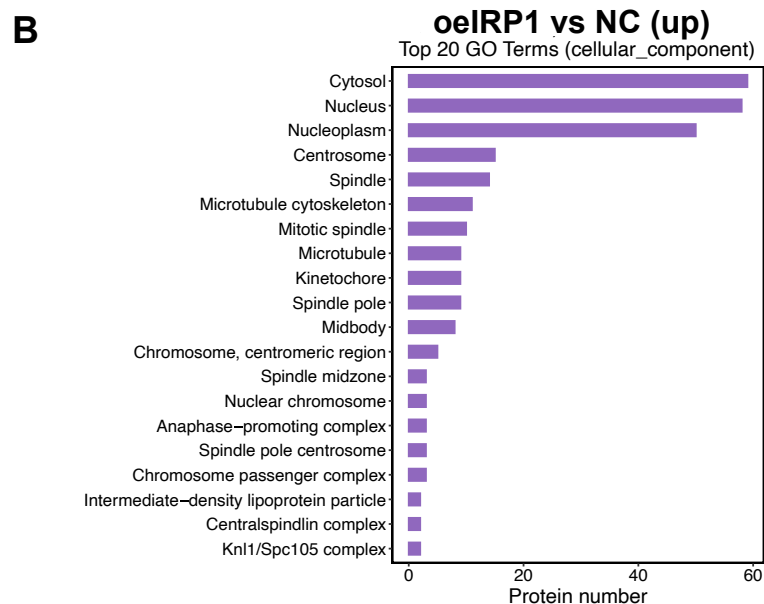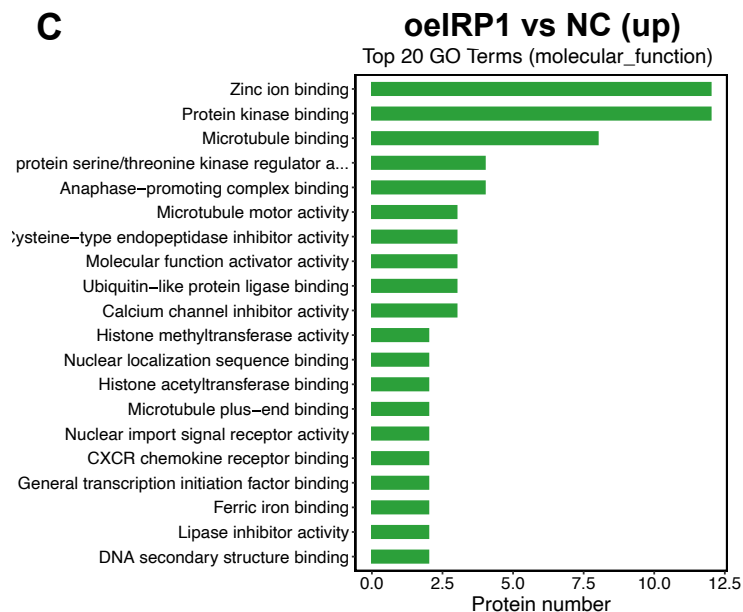

Supplement: Figure S3 — Principal component analysis and gene ontology analysis of PRO-DIA proteome (IRP1-overexpressed and control). (A) Principal component analysis of the IRP1-overexpressed and control groups in the PRO-DIA proteome. (B, C) Gene ontology analysis of the differentially expressed genes between the IRP1-overexpressed and control groups in the PRO-DIA proteome. [file mmc5.pdf]

# A Hemin(+)

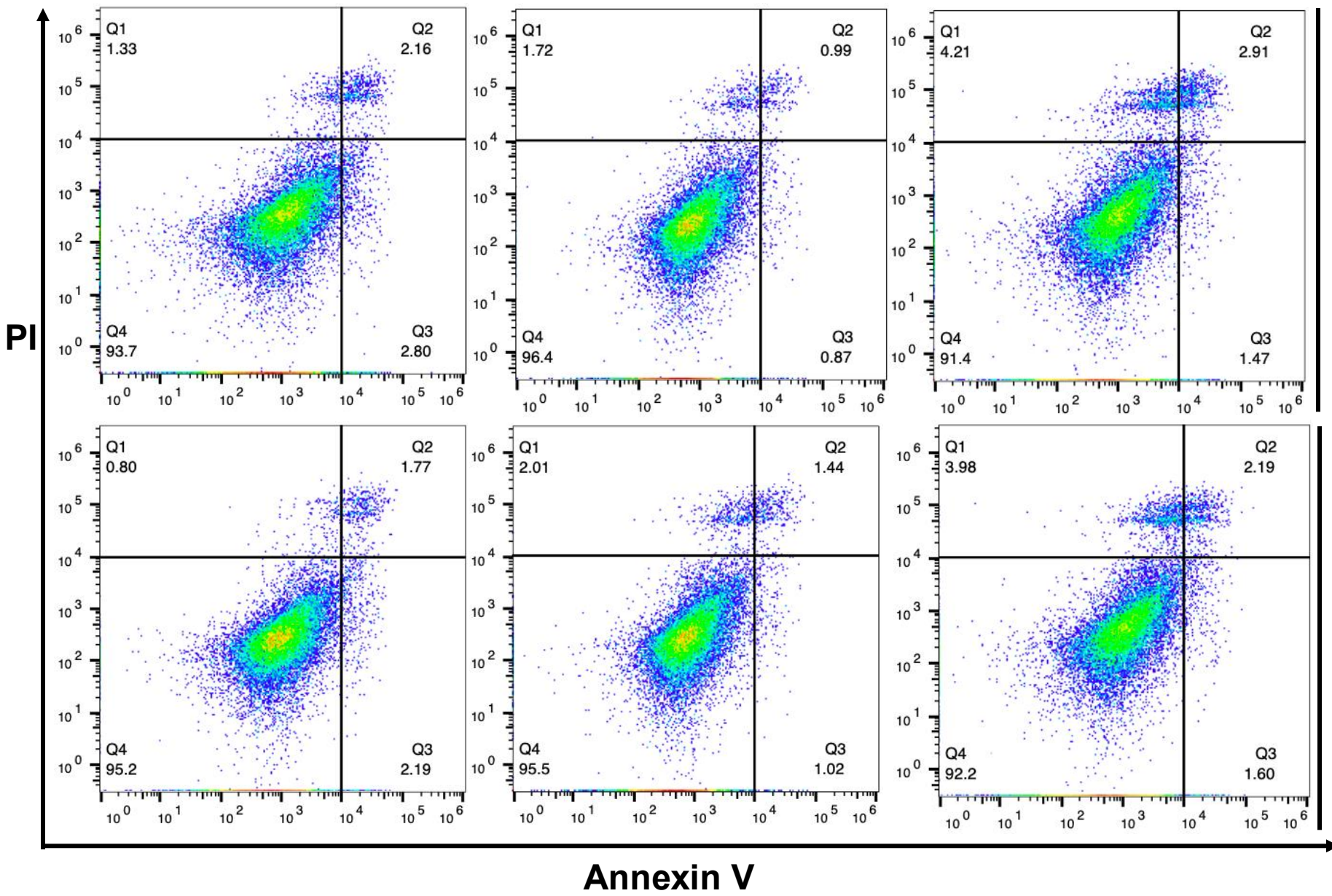

**Patu-8988**

**PANC-1**

## B

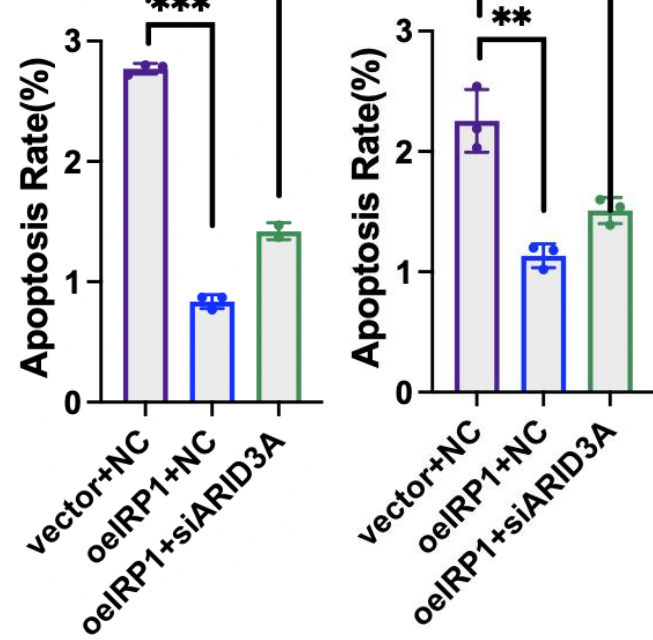

# C Hemin(+)

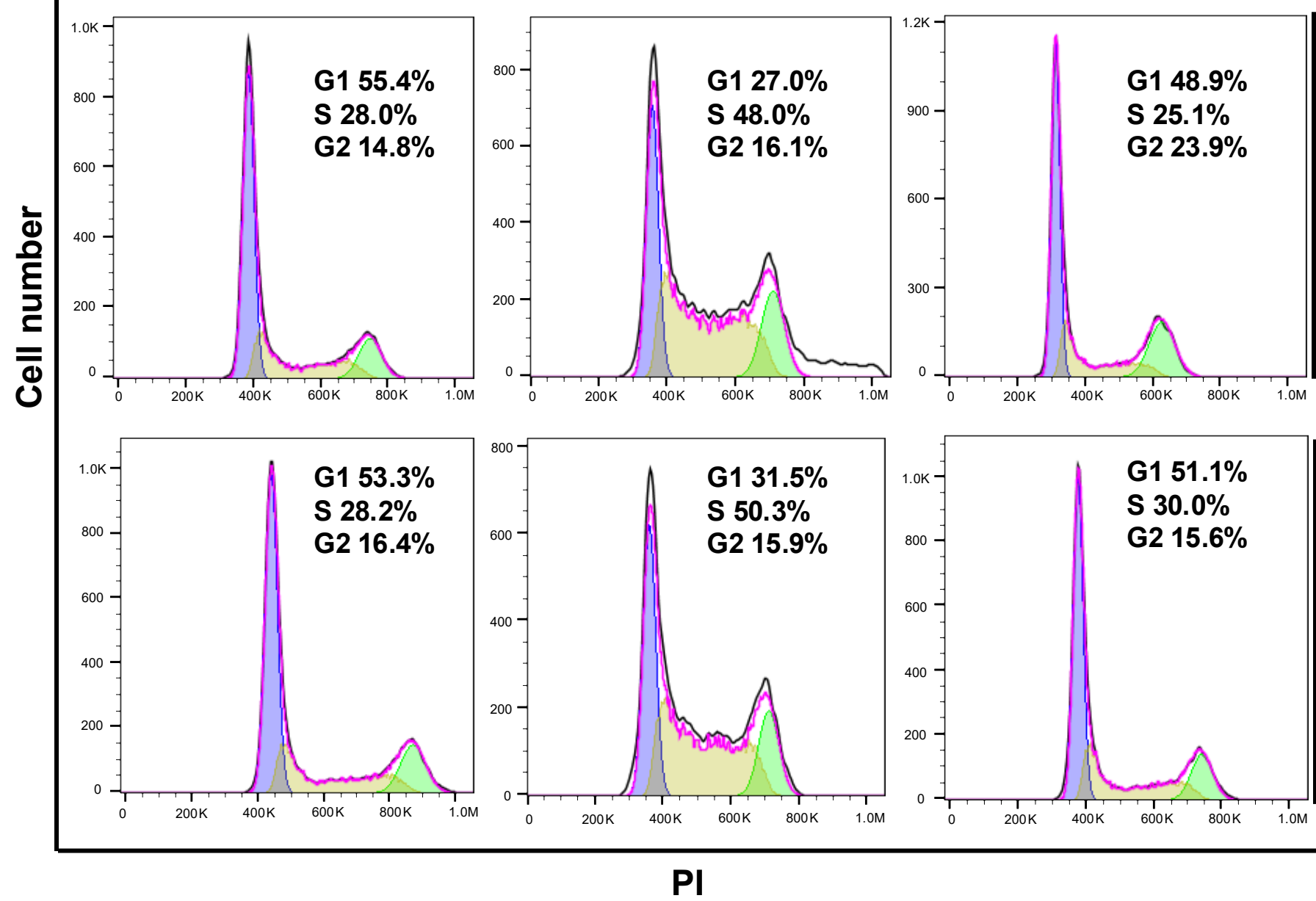

**Patu-8988**

**PANC-1**

## D

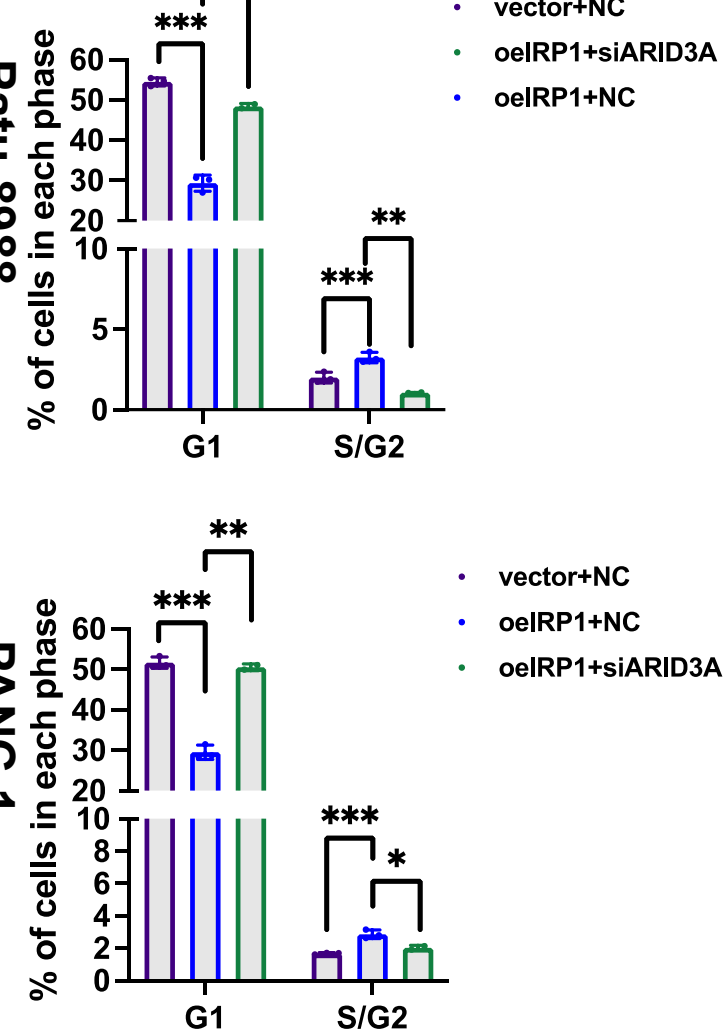

Supplement: Figure S4 — Regulation of holo-IRP1/ARID3A axis on pancreatic cancer cell apoptosis and cell cycle. (A, B) Apoptosis rate of pancreatic cancer cells with IRP1 overexpression and ARID3A silencing. (C, D) Cell cycle of pancreatic cancer cells with IRP1 overexpression and ARID3A silencing. [file mmc6.pdf]

**Library-construction**

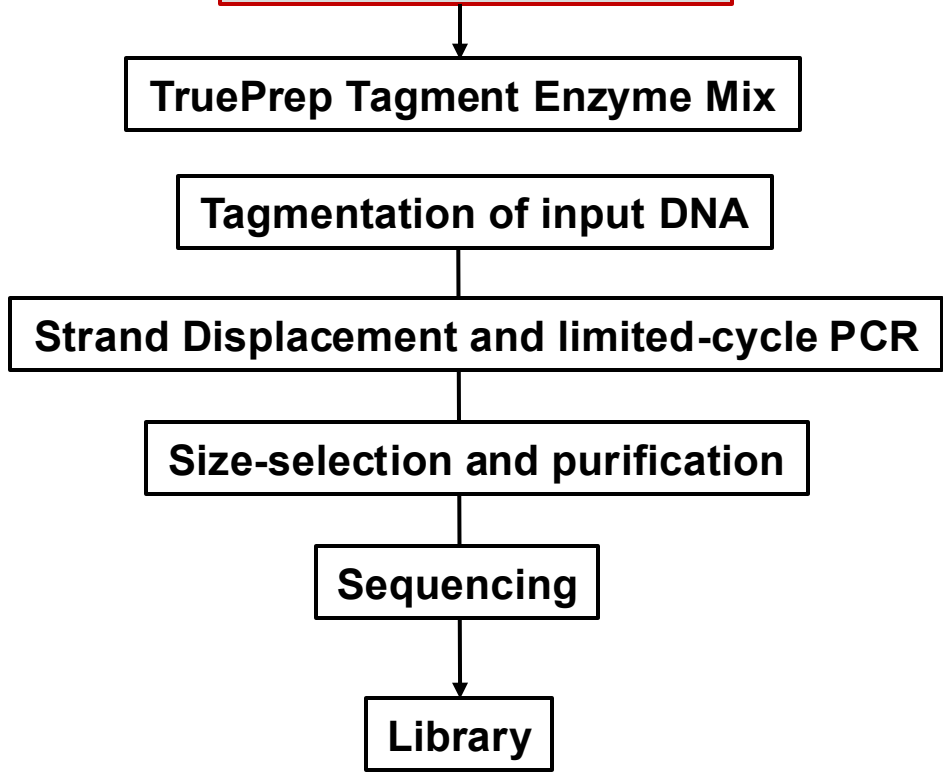

**Bioinformatics analysis**

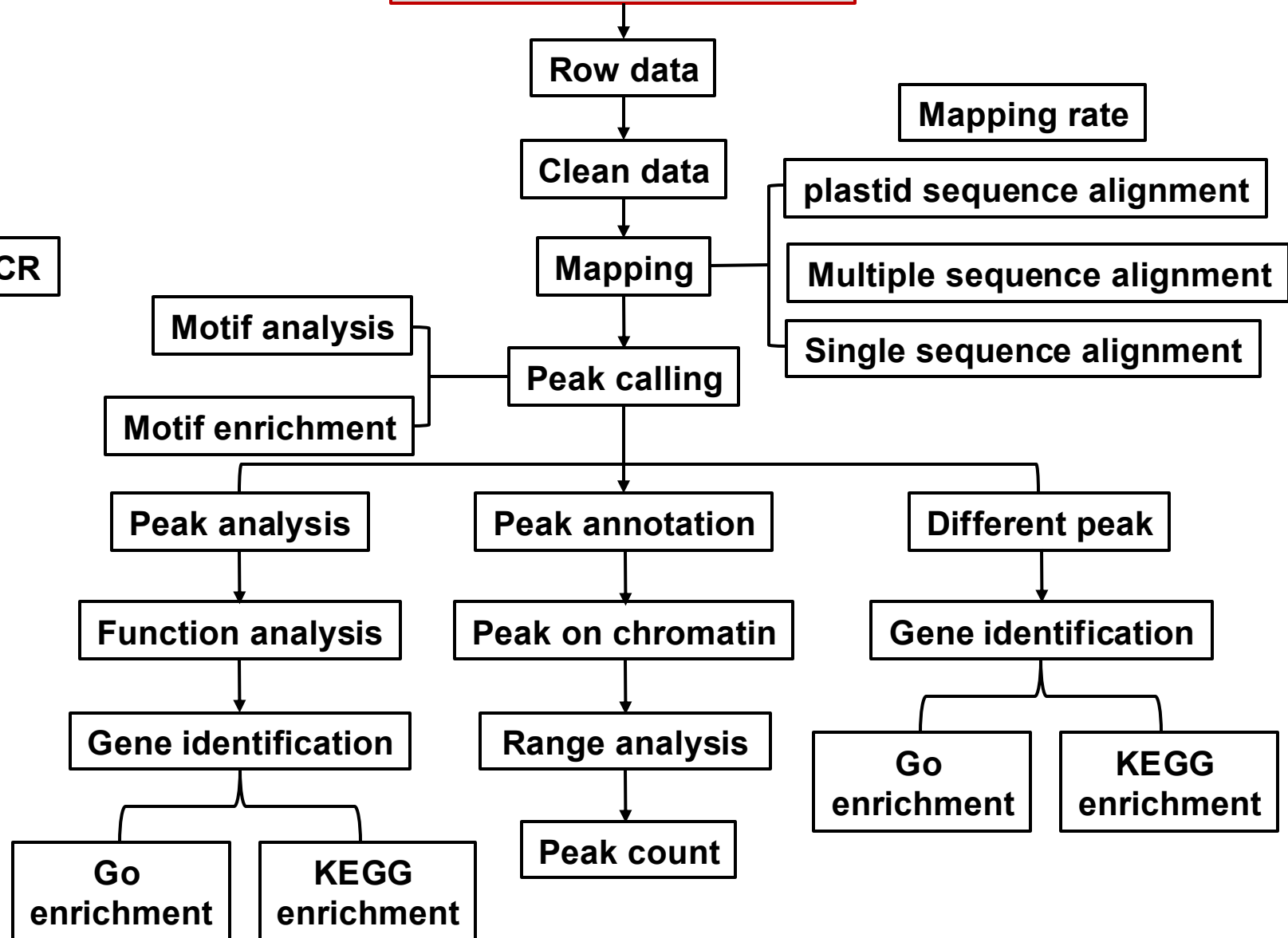

Supplement: Figure S5 — Workflow of ATAC sequencing analysis. [file mmc7.pdf]

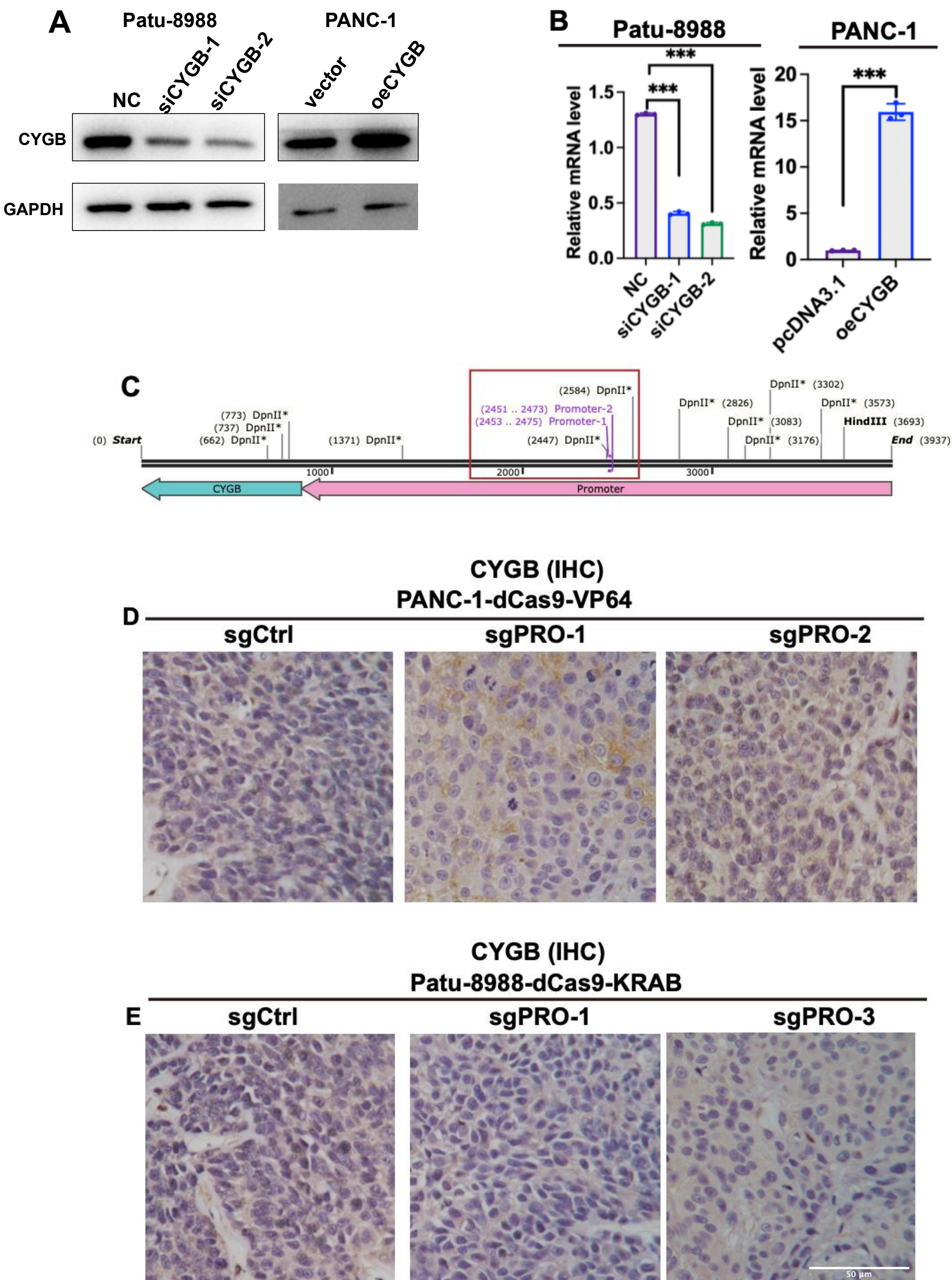

Supplement: Figure S7 — Transfection efficiency of siRNAs and plasmid of CYGB, design of CYGB promoter sgRNAs, and immunohistochemistry against CYGB. (A) The transfection efficiency of siRNAs and overexpression plasmid targeting CYGB was verified by western blotting. (B) Transfection efficiency of siRNAs and overexpression plasmid targeting CYGB was verified by quantitative reverse transcription PCR (∗∗∗P < 0.001). (C) Site pattern map of sgRNAs targeting the CYGB promoter region. (D, E) CYGB immunohistochemistry staining of xenograft tumor tissues. [file mmc9.pdf]
