# Supplementary material for: IRP1/ARID3A complex promotes pancreatic cancer chemoresistance by suppressing CYGB-related ferroptosis
Source: Genes Dis. 2025 Sep 24;13(5):101866. doi: 10.1016/j.gendis.2025.101866 (PMC13254595; doi:10.1016/j.gendis.2025.101866)
Supplement: Figure S6 — Luciferase analysis in PANC-1 and Patu-8988 cells. Relative luciferase intensity in PANC-1 and Patu-8988 cells transfected with corresponding designed plasmids. [file mmc8.pdf]

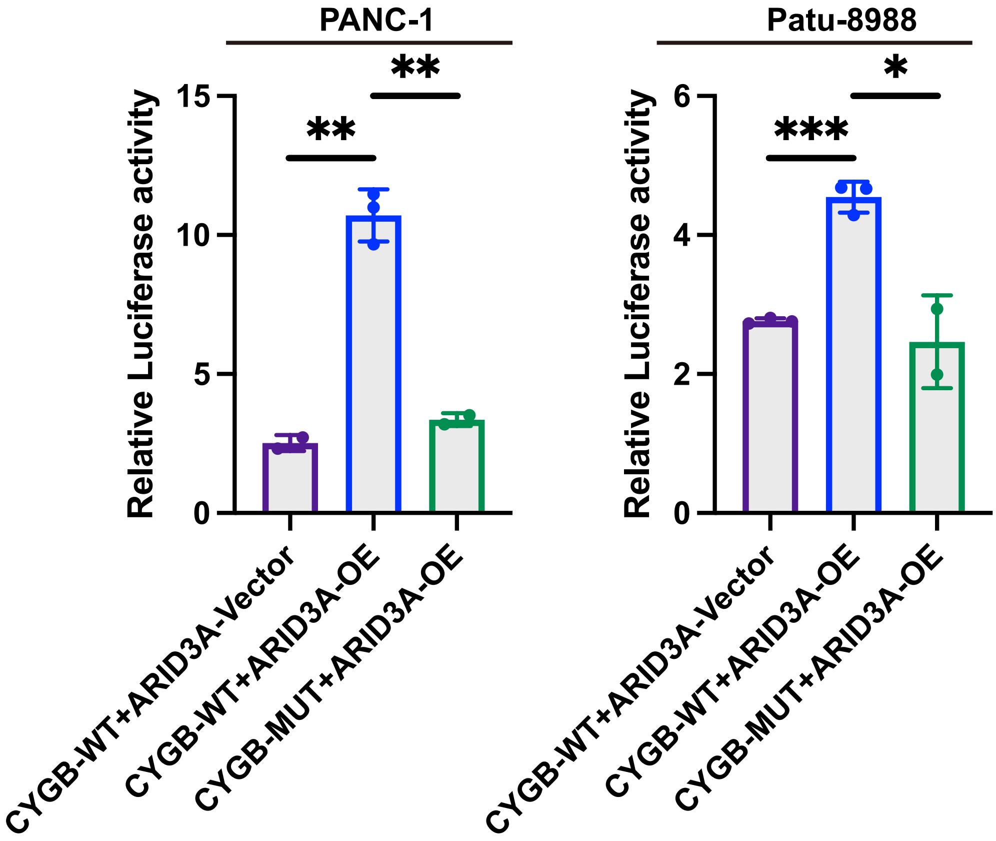
PANC-1
Patu-8988
Relative Luciferase activity
15
10
5
0
6
4
2
0
**
**
***
*
CYGB-WT+ARID3A-Vector
CYGB-WT+ARID3A-OE
CYGB-MUT+ARID3A-OE
